# A general data-driven framework for scalable electron tomography

**DOI:** 10.1093/nsr/nwag365

**Published:** 2026-06-16

**Authors:** Han Li, Wenting Cui, Huan Lei, Ziqi Chen, Yinpin Wei, Xuan Luo, Jiali Yang, Kuang Yu, Jia Li, Lin Gan

**Affiliations:** Institute of Materials Research, Tsinghua Shenzhen International Graduate School, Tsinghua University, Shenzhen 518055, China; Institute of Materials Research, Tsinghua Shenzhen International Graduate School, Tsinghua University, Shenzhen 518055, China; Institute of Materials Research, Tsinghua Shenzhen International Graduate School, Tsinghua University, Shenzhen 518055, China; Institute of Materials Research, Tsinghua Shenzhen International Graduate School, Tsinghua University, Shenzhen 518055, China; Institute of Materials Research, Tsinghua Shenzhen International Graduate School, Tsinghua University, Shenzhen 518055, China; Institute of Materials Research, Tsinghua Shenzhen International Graduate School, Tsinghua University, Shenzhen 518055, China; Institute of Materials Research, Tsinghua Shenzhen International Graduate School, Tsinghua University, Shenzhen 518055, China; Institute of Materials Research, Tsinghua Shenzhen International Graduate School, Tsinghua University, Shenzhen 518055, China; Institute of Materials Research, Tsinghua Shenzhen International Graduate School, Tsinghua University, Shenzhen 518055, China; Institute of Materials Research, Tsinghua Shenzhen International Graduate School, Tsinghua University, Shenzhen 518055, China

**Keywords:** atomic electron tomography, deep learning, multi-scale 3D imaging, depth-of-field

## Abstract

Electron tomography (ET) is crucial for determining the three-dimensional (3D) structure of materials in real space but challenging due to the inherent missing wedge, high dose, and limited depth-of-field. Although deep learning can address these challenges in principle, the scarcity of ET data severely limits its application. In this study, we propose a general data-driven ET reconstruction framework that uses extensive and readily available random high-entropy projections to construct large-scale datasets. By integrating both real structural priors and depth-dependent imaging physics, the framework enables high-quality 3D reconstruction independent of specific materials or resolutions. Using this strategy, we successfully determine the 3D atomic structure of a 13-nm Pt nanoparticle containing 52 138 atoms, achieving a root-mean-square displacement of 22.6 pm; the projection consistency error is significantly reduced, effectively expanding the depth-of-field limit of atomic-scale ET.

## Teaser

This study presents a big-data-driven electron tomography framework that learns from extensive real microscopy images, enabling 3D reconstruction across diverse materials while extending atomic-scale 3D imaging to thicker nanoparticles.

## INTRODUCTION

Electron tomography (ET) is an advanced characterization method for determining the three-dimensional (3D) structure of matter [1,2]. For inorganic specimens containing heavy elements, ET can be performed using an annular dark-field scanning transmission electron microscope (ADF-STEM), where image intensity monotonically increases with both thickness and atomic number (*Z*) [3–5]. Therefore, Radon-transform-based iterative reconstruction methods (IRM) [6–9] are commonly employed for ET reconstruction. However, traditional ET requires acquiring a large number of linear projections over a wide angular range at small intervals. Regular ET sample holders can only provide projection information within a finite angular range. To control acquisition time and irradiation dose, tomographic data acquisition inevitably involves under-sampling, thereby leading to a degraded reconstruction. Furthermore, the limited depth-of-field in microscope is particularly prominent at atomic resolution: atoms that are far from the focal plane cannot be imaged clearly [3,10]. These factors impede the application of ET to beam-sensitive materials, resulting in difficulties in resolving the accurate 3D atomic structures of large-sized materials thicker than ∼8 nm (Fig. S1).

Recently, deep learning (DL) has led to a paradigm shift in solving tomographic inverse problems through the development of numerous data-driven reconstruction algorithms [11–15], including post-processing [11,12], sinogram complement [13,14], fully learned [15], etc. However, data scarcity remains a major bottleneck limiting the application of DL methods in ET. Due to the complexity of ET experiments, the acquisition of 3D data is prohibitively expensive and time-consuming. For specimens that are beam-sensitive or thicker than the depth-of-field, the ground truth is inaccessible. To overcome these obstacles, a common class of approaches involves modeling material structures and generating training data through image simulations [11,16–18]. The effectiveness of such methods highly depends on the similarity between the training set and test data. However, the morphology and structure of real material systems are highly complex and often unknown, posing significant challenges to precise modeling. Moreover, the computational cost of simulations also increases rapidly with the spatial scale. Therefore, current data-driven ET remains mainly limited to a few specific materials [11,14], resulting in insufficient universality. In contrast, another class of methods avoids modeling specific structures but instead uses random complex data (e.g., natural images) to construct the training set, thereby guiding the neural network to learn the general principles for solving inverse problems. These methods, known as physical priors [19], have been widely applied to various imaging inverse problems such as ptychography [20] and holography [21]. However, due to the inherent information loss of tomography, sole reliance on a physical prior usually leads to sub-optimal reconstruction.

In this study, we propose a general strategy for generating large-scale ET training sets. Based on the encoding property of the projection process for 3D structural information, our method utilizes high-entropy, random electron microscopy (EM) images as data sources and incorporates non-ideal experimental effects to construct paired degraded datasets that integrate both structural and physical information. This enables robust ET reconstruction across different materials and resolutions, and is validated on experimental data of a wide range of complex real materials, eliminating the need for complex structural modeling, time-consuming simulations or expensive experiments, fundamentally overcoming the data scarcity. Furthermore, by integrating the depth evolution of defocus into the forward model, our method enables highly accurate and rapid atomic electron tomography (AET) reconstruction of thick specimens. As exemplified, we successfully reconstruct a 13-nm octahedral Pt nanoparticle from ordinary ADF-STEM tilt-series, with a root-mean-square displacement (RMSD) of 22.6 pm and a common atomic ratio exceeding 98%. The projection consistency error is significantly reduced to half of that achieved by traditional methods, especially the surface regions that are crucial for catalysis, thus enabling precise structure–property correlations across larger spatial scales.

## RESULTS

### Workflow of data-driven ET reconstruction

ADF-STEM is a typical incoherent imaging mode, and its object–image relationship can be expressed as a multi-slice form [3,22]:


(1) \begin{eqnarray*}
I( {x,\ y}) &=& \int P\left( {\boldsymbol r,\ z} \right)\ \otimes \ O\left( {\boldsymbol r,\ z} \right)\ {\mathrm{d}}z,\\
&&{\mathrm{with}}\ P\left( {\boldsymbol r,\ z} \right) = {\left| {{\mathcal{F}}^{ - 1}\left\{ {A\left(\boldsymbol k \right){e}^{ - i{\mathrm{\chi }}\left( \boldsymbol k \right)}} \right\}} \right|}^2.\\
\end{eqnarray*}

Here, $\otimes$ represents a two-dimensional (2D) convolution at the depth *z*, $O( {{\boldsymbol r},\ z} )$ is the object function of the specimen, which approximately corresponds to the square of the electrostatic potential of the specimen, and $P( {{\boldsymbol r},\ z} )$ is the point spread function (PSF). PSF is the intensity distribution of the electron probe in 3D real space [3,10,23], including all aberrations $\chi$ such as defocus, spherical aberration, astigmatism, and coma aberration, as well as the contribution of aperture $A$. The aim of ET reconstruction is to invert the 3D object function ${{O}}$ from projected images $I( {x,\ y} )$ at different angles. The ideal tomography requires a spatial-translation-invariant PSF and a sufficient number of projections; otherwise, it will lead to a complex nonlinear and ill-posed inverse problem.

**Figure 1.**
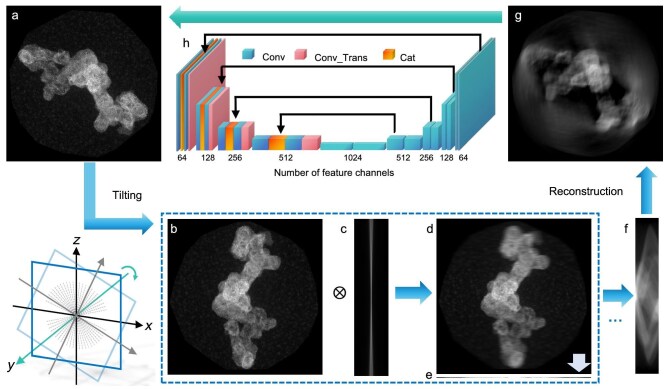
Workflow of data-driven ET reconstruction. (a) A random electron microscopic image used as the object function model. (b) Object function model rotated to different tilt angles. (c) The PSF of the ADF-STEM. (d) Object model after convolving with PSF at different depths (with the focal plane at the center). (e) Projection of (d) along the optical axis. (f) The projections (sinogram) of the object model (a). (g) The tomogram obtained by IRM. (h) The neural network model used for post-processing, which is trained with (g) as input and (a) as target (the structures and operation paradigms of neural networks can be arbitrary).

Given the scarcity of 3D images, constructing large-scale 3D tomographic datasets directly is impractical, necessitating appropriate approximations. In ET experiments, projections are typically acquired around a single tilt axis. Consequently, we regard the 2D digital image (Fig. 1a) as a pseudo-3D object with a thickness of 1 voxel along the tilt axis ($y$-direction), convolve it with the radial section $P( {r,\ z} )$ of the PSF at different depths $z$, and compute its projection (Fig. 1b–e). This approximation effectively overcomes data scarcity, preserves the sidelobe structures and probe spread caused by defocus, and significantly reduces the computational cost of processing large-sized 3D tomograms. According to the acquisition conditions, we further introduce the non-ideal factors such as missing wedge to precisely simulate the imaging process. By repeating the above steps, we obtain projections at different tilt angles (Fig. 1f). Subsequently, the IRM calculations are performed to generate the initial reconstruction (Fig. 1g). For simplicity, we adopt a standard 2D U-Net [24] convolutional neural network (CNN), which features an encoder–decoder structure with residual connections (Fig. 1h) and is trained to map the degraded tomograms (Fig. 1g) back to the original object models (Fig. 1a).

### Analysis of dataset for deep learning ET reconstruction

To explore the correlation between dataset attributes and tomographic reconstruction performance, we first apply the CNNs trained by our strategy to conventional ET which conforms to the classical linear projection approximation. For AET of small nanoparticles (<8 nm) and nanoscale ET of specimens hundreds of nanometers thick, the spatial inhomogeneity of the PSF can be neglected (Fig. S1). The 3D object to be reconstructed is actually the 3D potential function of the specimens, which is uniformly blurred by spherical aberration, thermal vibrations, and other factors. To accelerate data acquisition and reduce electron dose, we set the angular interval to 6° within ±66° range and employ a CNN to correct the artifacts arising from the missing wedge and insufficient data. Then, we employ three different datasets, that is, simulated random atomic tomograms, random experimental EM images, and random natural images [25], to train three CNN models, denoted as Tomo-CNN, EM-CNN, and ImgNet-CNN (Section S1.1 and S1.2, Figs S2 and S3). Each dataset contains 10^6^ data samples.

**Figure 2.**
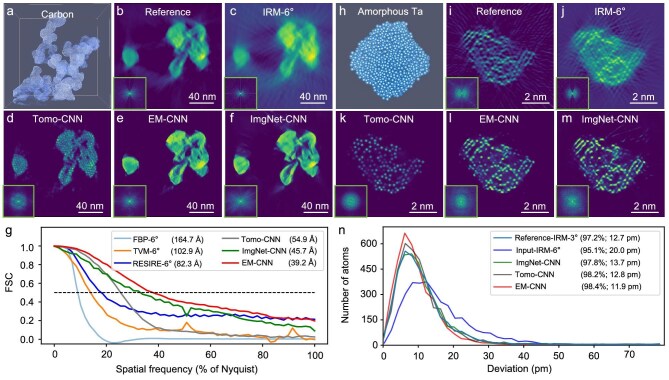
Multi-scale ET reconstruction performance of deep learning models trained on different datasets. (a) 3D tomogram of an EC300J nanoporous carbon material (412^3^ voxels, with a voxel size of 6.977 Å) reconstructed by EM-CNN at 6° intervals. (b and c) The tomograms of carbon reconstructed by IRM (RESIRE) from tilt series at 2° (b) and 6° (c) intervals. The lower left shows their Fourier amplitude in logarithmic scale. (d–f) The tomograms of carbon reconstructed by CNNs trained on different datasets. (g) Fourier shell correlation curve of carbon materials, with the 3D resolution estimated by the 0.5 criterion in legend. (h) Atomic model of amorphous Ta nanoparticles. (i and j) The tomograms of Ta nanoparticles reconstructed by IRM from tilt-series at 3° intervals (i) and 6° intervals (j). (k–m) The tomograms of Ta nanoparticles reconstructed by CNNs trained on different datasets. (n) Histogram of Ta atomic coordinate deviations. The common atomic ratio and RMSD are shown in legend.

We first evaluate the performance using a nanoscale ET tilt series of EC300J carbon materials. Due to the complex internal pore structure of this specimen (Fig. 2a and b, Fig. S4), it is difficult to construct a dedicated training set. One can see that the severe under-sampled artifacts cause the pores and surfaces to be obscured at 6° intervals (Fig. 2c). After CNN augmentation, the artifacts in the tomogram can be removed, and the missing information in Fourier space is also recovered (Fig. 2d–f). We adopt the Fourier shell correlation (FSC) coefficient in reciprocal space as the primary performance metric, and estimate the 3D resolution by cross-validation on multiple independent experimental datasets (Section S1.3 and S1.4). As shown in Fig. 2g, due to the information loss, the FSC decays rapidly even using the state-of-the-art IRM algorithm real-space iterative reconstruction [6] (RESIRE), and the resolution of the 3D tomogram is considerably lower than that of the projected images. Similarly, the training set of Tomo-CNN provides inaccurate structural prior information to recover the high-frequency details, which also leads to fast decay of the FSC. In contrast, the FSC curve of EM-CNN better matches the ideal natural signal. It decreases monotonically and gradually, extending smoothly to higher spatial frequencies. The result of ImgNet-CNN lies between these two cases. Both 3D segmentation and local resolution validation (Fig. S4) confirm that EM-CNN exhibits the highest projection and reconstruction consistency in real space among all training sets. We further performed blind tests using nanoscale ET tilt series of Co_3_O_4_ nanosheets (voxel size: 224 Å, see Fig. S5), MnO_2_ nanowires (voxel size: 158.6 Å, see Fig. S6), as well as publicly available datasets of a hyperbranched Co_2_P nanocrystal [26] (voxel size: 7.1 Å, see Fig. S7) and a nanoporous PtCu catalyst [26] (voxel size: 3.8 Å, see Fig. S8). EM-CNN consistently demonstrates stable and optimal performance across changes in resolution, image size, and materials, which is helpful for low-dose and fast 3D imaging of electron beam sensitive materials with unknown structure (Table S1). We also conducted a quantitative comparison of EM-CNN with the previously reported self-supervised IsoNet method [27], which is generally applied to biological specimens with sufficient learnable orientations. As shown in Fig. S9 and discussed in Section S2, IsoNet fails to stably reconstruct material specimens that lack structural redundancy, whereas EM-CNN achieves better performance.

We further assess CNN performance at the atomic scale using multi-slice simulated tilt series of an amorphous Ta model [28] (Section S1.5–S1.8, Fig. 2h). Missing wedge and limited data lead to blurred and elongated atomic features (Fig. 2i and j), which are substantially suppressed after CNN augmentation (Fig. 2k–m). Tomo-CNN shows the best visual fidelity due to its accurate atomic priority, while EM-CNN and ImgNet-CNN slightly distort atomic shapes but markedly improve atomic consistency. After atomic tracing and refinement, EM-CNN achieves an RMSD of 11.9 pm with a common atomic ratio above 98% (Fig. 2n, Table S2). Batch tests confirm that EM-CNN consistently outperforms ImgNet-CNN, which lacks the prior of the microscopic world (Fig. S10) and yields results comparable to Tomo-CNN trained on dedicated datasets, demonstrating its robustness for structures beyond specific modeling assumptions.

**Figure 3.**
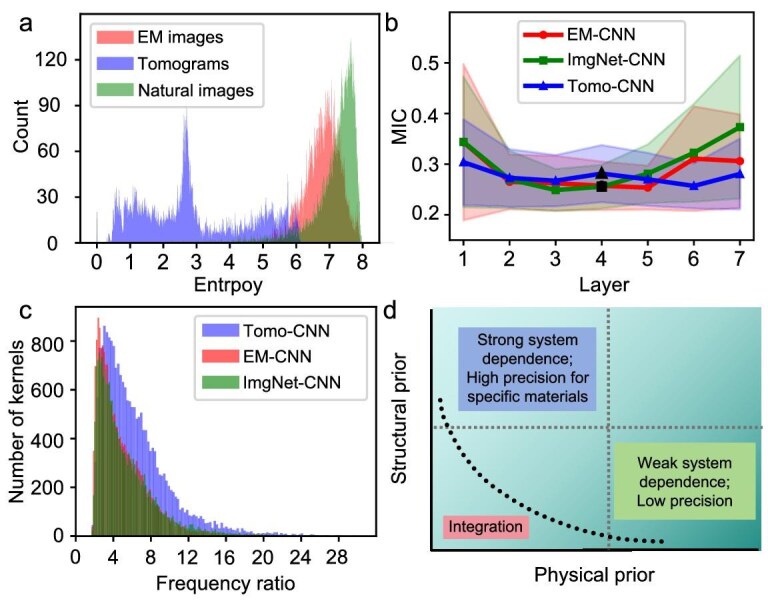
Effect of dataset entropy of deep learning models. (a) Shannon entropy histograms of different datasets (statistically obtained from 10 000 random samples). (b) Averaged MIC and standard deviation for different CNNs. Black represents the bridge layer. (c) Ratio of high- to low-frequency components of the convolutional kernels in decoders from different CNNs. (d) Schematic of the optimal frontier curve between the physical and structural prior in the ET inverse problem.

To analyze the attributes of different training sets and their impact on CNNs, the Shannon information entropy is used as a quantitative metric [29] (see Section S1.9). Entropy is a measure of the amount of information. A higher entropy indicates a more uniform distribution and a broader coverage, thereby reflecting greater statistical diversity. The statistical results in Fig. 3a show that both EM images and natural image datasets have significantly higher entropy than electron tomograms. For the vast majority of them, entropies exceed 6 bits, covering a wide range of content and indicating weak priors. Therefore, to reduce loss, neural networks must learn universal principles (that is, physical priors). In contrast, low-entropy tomograms cannot ensure this; as shown in Fig. S3, Tomo-CNN converges rapidly on the atomic tomogram training set but performs poorly in nanoscale tomograms outside the training distribution (Fig. 2a–g and Figs S4–S8). This implies that CNN can minimize losses by memorizing and replicating features of specific datasets.

To verify the above inference, we first analyse the feature map correlation of the middle layer of the CNN on the validation set (Fig. 3b), which is measured by the maximal information coefficient [30] (MIC, see Section S1.10). It is found that the MICs of EM-CNN and ImgNet-CNN gradually decrease from shallow to deep layers during the encoding stage (1$\to$4 layers), indicating that the models progressively extract more independent higher-level features, while Tomo-CNN remains basically unchanged. In the bridge layer with the strongest independence feature, the average MICs of EM-CNN and ImgNet-CNN are significantly lower than those of Tomo-CNN. This suggests that CNNs trained on high-entropy datasets can learn more stable and generalizable patterns, thereby enabling efficient information compression [31]. In Fig. 3c, we further calculate the ratio of high- to low-frequency components in the convolution kernels of the decoder (see Section S1.11), as it is the key operation to recover the compressed information. The ratio of high-frequency components in the convolution kernels of Tomo-CNN is significantly higher than in EM-CNN and ImgNet-CNN trained on high-entropy data. This indicates that the low-entropy training set causes the CNN to tend to memorize high-frequency details, resulting in poor generalization and robustness [32]. Collectively, through efficient information compression and generating low-frequency convolution kernels, the high-entropy datasets fundamentally inhibit CNN from counterfeiting structural details, this is the fundamental reason for the strong generalization ability of EM-CNN and ImgNet-CNN.

**Figure 4.**
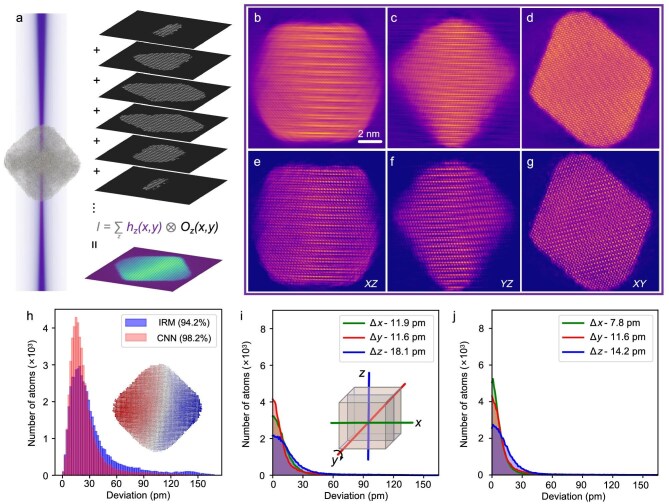
Defocus-corrected 3D reconstruction of a large-sized Pt nanoparticle. (a) ADF-STEM imaging model for thick specimens. (b–d) Three orthogonal cross-sections of the tomogram reconstructed by IRM, serving as input to the CNN. (e–g) Three orthogonal cross-sections of the tomogram processed by the CNN. (h) Histogram of atomic deviations, with common atomic ratio shown in legend. (i and j) Histograms of atomic deviations in $x,\ y$, and $z$ directions before (i) and after (j) the CNN augmentation, with the mean deviations shown in legend.

However, due to the complexity of real material systems and the inherent information loss in the tomographic inverse problem, relying solely on structural characteristics of the specific materials or physical priors (universal imaging laws) is not an optimal solution. It is necessary to seek a balance between the two priors (Fig. 3d). As shown in Fig. 4a, from the imaging perspective, an EM image represents a superposition of a series of low-entropy tomograms encoded by the projection operation. Consequently, EM images consistently exhibit higher entropy than tomograms (Fig. 3a and b, Fig. S11), while still containing real structural priors from the microscopic world. Therefore, when the EM-CNN trained on random high-entropy EM images is applied to low-entropy tomograms, its fidelity is better than ImgNet-CNN. Most importantly, compared with expensive and generalization-limited tomograms, the acquisition cost of random projected images is almost negligible. As an effective integration of physical and structural priors, the model is ultimately trained using random EM images.

### Application in AET with extended depth-of-field

We extend the data-driven method to AET of large-sized specimens. In addition to the data insufficiency caused by increased thickness [33], AET is further constrained by the depth-of-field. The top and bottom regions of the thick specimen cannot be focused simultaneously, leading to varying defocus at different depths (Fig. 4a, Fig. S1). This effect violates the linear projection assumption. Therefore, current Radon-transform-based AET remains confined to thin specimens. For AET of large-sized specimens thicker than 8 nm, the currently reported projection consistency error approaches 10% [18,28,34] and the RMSD exceeds 30 pm [18,34,35]. Bridging this gap is of great practical importance, as it enables a more accurate correlation between atomic structures and properties, while also mitigating the changes caused by thinning during specimen preparation.

To address these challenges, it is necessary to modify the forward model [36,37]. Within the optimal aperture, astigmatism and coma can be approximately neglected. Meanwhile, spherical aberration and thermal vibrations can be incorporated into the object function $O^{\prime}$. Therefore, the object–image relationship for thick specimens at atomic resolution can be simplified to a linear projection of the object function $O{^{\prime}}_z( {x,\ y} )$ convolved with the defocus kernel ${h}_z( {x,\ y} )$ [3]:


(2) \begin{eqnarray*}
I( {x,\ y} ) &=& \mathop \sum \limits_z {h}_z( {x,\ y})\ \otimes \ O{^{\prime}}_z ( {x,\ y}),\\
&&\!\!\!\!\!\!\!\!\!\!\!\!\!\!\!\!\!\!\!\! {\mathrm{with}}\quad {h}_z( {x,\ y} )\\
&=& {\big| {\mathcal{F}_{xy}^{ - 1}\big\{ {A\left( {{k}_x,{k}_y} \right){e}^{ - i\pi \lambda ( {k_x^2 + k_y^2} )\Delta {f}_z}} \big\}} \big|}^2.\\
\end{eqnarray*}

Here, $\lambda$ denotes the wavelength and ${\mathrm{\Delta }}{f}_z$ is the distance between the depth $z$ and the focal plane. Based on this, to construct the training set, we first apply the ${h}_z$ to object function $O^{\prime}$ at different depths to incorporate the depth dependence of the electron probe, and then compute their projections (Fig. 1b–f). A focal plane fluctuation of ±2 nm was added to simulate the random defocus deviation of the experiment. The remaining procedures are identical to those described above. For simplicity, EM-CNN with defocus correction is hereafter referred to as CNN.

After training, we apply the CNN to AET experimental data of a standard octahedral Pt nanoparticle with a maximum thickness of ∼13 nm along the optical axis. At this thickness, half of the particle cannot be focused (Fig. S1). To protect the beam-sensitive surface, we only collected 48 projections within ±70° range at a single focal depth (Fig. S12) and performed a preliminary reconstruction via IRM, where the atomic boundaries are nearly indistinguishable (Fig. 4b–d). Then we perform CNN augmentation, which requiring only 4500 MiB of memory and takes only 20 s on an ordinary GPU (RTX 2080Ti), and the clear atomic profiles and lattice structures is recovered (Fig. 4e–g, Fig. S13), even though the random EM images contain no explicit information about the Pt atomic potential. From the CNN tomograms, we identify 52 138 atoms, and after removing the surface atoms [34], 95.6% of them exhibit FCC ordering. In comparison, even using the state-of-the-art algorithm RESIRE, only 89.2% atoms exhibit FCC ordering. The atoms in the thicker regions were inconsistent with structural priors (Fig. S14).

To evaluate the uncertainty of atomic coordinates, we split the experimental data into two independent tilt series and assess their reconstruction consistency through cross-validation [38] (see Section S1.6). The RMSD of CNN reduced to only 22.6 pm with a common atomic ratio of 98.2% (Fig. 4h, Table 1), and the atomic deviations are mostly at the sub-pixel level. Sensitivity analysis shows that the normalized mean absolute error for strain measurement will reduce ∼4.3% [39]. Further analysis of the paired atomic displacement in three orthogonal directions (Fig. 4i and j) indicates that the CNN effectively corrects the atomic deviations along the $z$ and $x$ directions with the worst resolution, achieving obvious improvement compared to the IRM based on linear projection. Since the defocus kernels within ±6.5 nm resemble a series of depth-dependent Gaussian kernels with separable variables, the 2D approximation does not improve or introduce additional error along the tilt axis ($y$-direction) with the best intrinsic resolution.

**Table 1.** Quantitative analysis of reconstruction performance of a 13-nm Pt nanoparticle.

Methods	*R*_1_-factor error	*R*_F_-factor error	RMSD (${\mathrm{pm}}$)	Common atomic ratio	FCC ratio	Local lattice constant (Å)
CNN (defocus corrected)	5.32%	20.72%	22.6	98.2%	95.6%	3.918 ± 0.053
CNN (linear projection)	9.49%	39.51%	27.5	97.2%	87.9%	3.888 ± 0.068
IRM (RESIRE)	9.23%	40.20%	28.9	96.3%	89.2%	3.884 ± 0.065
IRM (SART)	11.48%	47.85%	32.6	94.2%	78.0%	3.869 ± 0.085
IRM (GENFIRE)	11.35%	56.31%	38.3	92.3%	70.2%	3.888 ± 0.091

**Figure 5.**
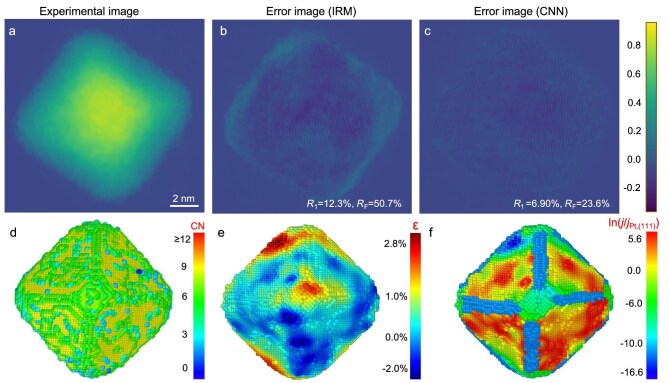
Surface structure analysis and application of a large-sized Pt nanoparticle. (a) Representative experimental image (43°). (b and c) Error images obtained by subtracting the computed projection of the IRM-reconstructed atomic structure (b) and the CNN-reconstructed atomic structure (c) from the experimental projection, respectively. (d) Visualized coordination numbers of the CNN structure. (e) Volumetric strain $( {{a}_{{\mathrm{loc}}} - {a}_{{\mathrm{ref}}}} )/{a}_{{\mathrm{ref}}}$ of the CNN structure, where ${a}_{{\mathrm{ref}}}$ is the bulk Pt lattice constant, and ${a}_{{\mathrm{loc}}}$ is the local lattice constant. (f) Visualized theoretical ORR activity ${\mathrm{ln}}( {j/{j}_{{\mathrm{Pt}},( {111} )}} )$, where $j$ is the current density.

To visualize the improvement, we use the 43° projection as an example, which flattens the thickest area of the particle onto a 2D projection (Fig. 5a, Figs S15 and S16). The IRM-reconstructed structure and its computed projection exhibit significantly higher consistency errors in the poorly focused surface regions, due to the inaccurate forward model (Fig. 5b). After CNN augmentation, the errors are significantly reduced (Fig. 5c), which is crucial for catalysis and other surface-sensitive applications. We employ the *R*_1_-factor and the *R*_F_-factor error as metrics (specifically, these correspond to the normalized L1 norm error between the computed projection of the atomic structure and the experimental projection, in real space and Fourier space, respectively [38,40], see Section S1.6). The errors of the CNN-reconstructed structure are approximately half that of the IRM or CNN without defocus correction (Table 1).

We further evaluate the rationality of the surface atoms using the coordination numbers (CN) as the descriptor, as shown in Fig. S17. The surface of IRM structure contains numerous atoms with abnormally high or low CN, with a value of $( {{\mathrm{8}}{\mathrm{.08\ \pm \ 2}}{\mathrm{.28}}} )$. In contrast, the CNN structure is more consistent with the general characteristics of FCC crystals (Fig. 5d). The CN of the steps, corners and edges range from 5 to 7, whereas the outer surfaces generally have a CN of 8–9, with a value of $( {{\mathrm{8}}{\mathrm{.03\ \pm \ 1}}{\mathrm{.45}}} )$. Further ablation studies about the PSF (Figs S18 and S19), multiple scattering (Fig. S20), training set size (Fig. S21), electron dose (Fig. S22), residual aberrations (Fig. S23), contrast mechanism (Fig. S24), and image stitching (Fig. S25) are shown in the Supplementary Information. The CNN with defocus correction avoids the complexity of iterative inversion and effectively compensates for the information loss caused by defocus, outperforming depth-dependent IRM methods (Section S2, Fig. S26). The CNN method remains robust to residual aberrations but is susceptible to dynamic diffraction effects, such as channeling and multiple scattering. Zone axes should be avoided in experiments, and the credibility of atoms should be carefully evaluated through cross-validation (Section S3).

For the catalyst specimen, an accurate surface structure enables us to directly predict the overall reactivity of the nanoparticle based on the experimentally measured facet types and atomic strain [41] (Section S1.12, Fig. S27). As shown in Fig. 5e and f, although the oxygen reduction reaction (ORR) activity of the particle mainly depends on the type of facet, it also shows a strong dependence on strain. The data-driven defocus-corrected AET, serving as a bridge between theoretical mechanisms at the atomic scale and experimental measurements at the macroscopic scale, allows direct correlation and mutual validation between them.

In experiments, defocus can also be suppressed by reducing the convergence semi-angle [3] (that is, aperture $A$). However, this results in an overall resolution degradation. We reconstruct two disordered amorphous Pd nanoparticles from the publicly available experimental datasets acquired using a low convergence semi-angle [28]. The error of the CNN-reconstructed structure is also obviously lower than traditional Radon-based methods (Figs  S28 and S29, Table S3). Collectively, we believe our method matches the imaging physics of large-sized specimens more accurately.

## DISCUSSION

We propose a general data generation paradigm for deep learning ET reconstruction, overcoming the data scarcity of the large-sized complex real materials. Our findings highlight that high-entropy projected images can effectively drive neural networks to learn the general physical principles of tomographic inverse problems, while incorporating real structural information to achieve a balanced prior integration. For atomic-scale 3D reconstruction of large-sized materials, by introducing the imaging physics of defocus, we overcome depth-of-field limitations and achieve high-precision reconstruction of Pt nanoparticles with thicknesses up to 13 nm. This strategy can be generalized to other imaging inverse problems that suffer from data insufficiency and require prior information, and promote the further application of artificial intelligence in materials characterization.

## MATERIALS AND METHODS

### Collection of data sources

Random atomic tomograms: According to the method reported in literature [11], the random atomic tomograms are obtained by convolving the Gaussian kernel with 3D atomic potential of a random-shaped nanoparticle. The sizes of the random nanoparticles are all between 4 and 6 nm, as modeling and simulation of large-scale material systems with tens of thousands of atoms is time-consuming.

Random electron microscopic images: Warwick electron microscopy dataset [42] (containing 37 035 images, with image sizes of 1024^2^ and 2048^2^), virus electron microscopic image dataset [43] (containing 22 virus classes, 1245 images with pixel sizes ranging from 0.26 to 5.57 nm and image size ranging from 1376 × 1032 to 2048^2^), metal catalyst dataset [17] (containing 189 images with size of 2048^2^), and projected images reported in literature [11,18,28,35,38–41,44–49]. The above datasets are all public and available. We use them as the data source for EM-CNN and the defocus corrected AET.

Random natural images: The large natural image dataset ImageNet [25] contains over 20 000 types of objects and more than 14 million images. We randomly select a part of them as the data source for ImgNet-CNN.

### ET data acquisition

The detailed process of specimens preparation is provided in the Section S4. The ET tilt-series of EC300J nanoporous carbon is collected on a Spectra 300 double-aberration-corrected STEM with Fischione 2020 holder. The acceleration voltage was 200 keV, the convergence semi-angle was 25 mrad, and the detector inner and outer semi-angles was 39–200 mrad. A total of 50 projections were acquired over a tilt range from −72° to +68°. The images had a pixel size of 697.7 pm, with one image acquired at each angle and a dwell time of 5 μs.

The AET tilt series of the Pt nanoparticle was collected on the same electron microscope. The acceleration voltage was 200 keV, the convergence semi-angle was 30 mrad, the detector inner and outer semi-angles were 62–200 mrad, and the pixel size is 31.85 pm. All images were captured under focused conditions. During the acquisition of the tilt-series, we adjusted the A1 and B2 astigmators of the microscope at each angle using other nanoparticles near the target particle to minimize astigmatism and achieve optimal imaging conditions. Therefore, the impact of astigmatism on reconstruction is relatively small. A total of 48 projections were acquired over a tilt range from −68° to +70°. To minimize sample drift, the sample was allowed to stabilize for one minute before acquiring each tilt. At each tilt angle, 10 images were acquired with a dwell time of 2 μs per pixel and then combined after drift correction. The electron beam current was 10 pA, and the total electron dose was 6.21 × 10^5^ e/Å^2^.

## Supplementary Material

nwag365_Supplemental_File

## Data Availability

The code, pre-trained model, tomographic data (tilt-series, tomograms, and atomic coordinates) and examples of this study are publicly available on GitHub: https://github.com/Thu-SIGS-EmLab/EM-CNN and repository: https://zenodo.org/records/20125876.
