# Supplementary material for: A general data-driven framework for scalable electron tomography
Source: Natl Sci Rev. 2026 Jun 16;13(14):nwag365. doi: 10.1093/nsr/nwag365 (PMC13397574; doi:10.1093/nsr/nwag365)
Supplement: nwag365_Supplemental_File [file nwag365_supplemental_file.pdf]

Supplementary information for

A General Data-Driven Framework for Scalable Electron Tomography

Han Li[1,†], Wenting Cui[1,†], Huan Lei[1], Ziqi Chen[1], Yinpin Wei[1], Xuan Luo[1], Jiali Yang[1], Kuang Yu[1], Jia Li[1,*] and Lin Gan[1,*]

[1]Institute of Materials Research, Tsinghua Shenzhen International Graduate School, Tsinghua University, Shenzhen 518055, China

***Corresponding authors.** E-mails: lgan@sz.tsinghua.edu.cn; li.jia@sz.tsinghua.edu.cn

[†]Equally contributed to this work.

This PDF file includes:

Supplementary Information
Figures S1 to S29
Tables S1 to S3

Supplementary Information Section 1: Computational Details

1.1 Construction of training sets First, we converted the images into square grayscale images. To avoid artifacts generated by the edge regions, we created a random convex hull located within the tangent circle of the image as a mask, retaining only the internal pixels and setting the rest to 0. Then, we performed forward projection through Eq. (2). Specifically, first the object function $\boldsymbol{O} \in \mathbb{R}^{N \cdot 1 \cdot N}$ is rotated to the corresponding tilt-angle θ, then the physical information of the imaging system is introduced by multiplying it with the transfer function H_z, and the projection is performed by linear summation:

$$I(x, y;\ \theta) = \sum_{z} \mathcal{F}^{-1}\left\{H_z\left(k_x, k_y\right) \cdot \mathcal{F}\{O'_z(x, y)\}\right\} \text{ , with } \boldsymbol{O'} = \boldsymbol{R_\theta O} \tag{1}$$

Here, $\boldsymbol{R_\theta}$ represents the operation of rotating $\boldsymbol{O}$ to the tilt-angle θ (around the y-axis). Finally, the input of CNNs is obtained through IRM calculation. The fast IRM algorithm SART is applied for iterative solving[1, 2]:

$$\boldsymbol{O}^{(k+1)} = \boldsymbol{O}^{(k)} + \frac{1}{nN}\boldsymbol{A}^{\mathrm{T}}\boldsymbol{D}^2\left(\boldsymbol{I} - \boldsymbol{A}\boldsymbol{O}^{(k)}\right), \boldsymbol{D} = \operatorname{diag}\left\{\frac{1}{\|\boldsymbol{a}_1\|}, \frac{1}{\|\boldsymbol{a}_2\|}, \ldots, \frac{1}{\|\boldsymbol{a}_{nN}\|}\right\} \tag{2}$$

Here, $\boldsymbol{a}_i$ represents the i-th row of Radon projection operator $\boldsymbol{A} \in \mathbb{R}^{(n \cdot N \cdot 1) \times (N^2 \cdot 1)}$, $\boldsymbol{I} \in \mathbb{R}^{n \cdot N \cdot 1}$ is the tilt-series. The advantage of SART lies in its rapid convergence, making it suitable for synthesizing large datasets. Then, these random images were cut into 256×256 patches through a sliding window, which are used as the input and target, respectively. Each of the three training sets contains 10^6 pairs of samples. The validation set is composed of publicly available real experimental ET datasets[3]. Due to the scarcity of ET data, we also performed augmentation by randomly scaling and flipping the images.

For the AET of thick specimens, to avoid unnecessary errors introduced by size changes, we converted all random EM images into 512×512 patches through interpolation and sliding windows, and constructed a training dataset of 5×10^5 pairs of images, of which 1/10 were used as the validation set.

1.2 Training and inference of the neural network The aim of the neural network $f_{\boldsymbol{\theta}}: \mathbb{R}^{N^2} \rightarrow \mathbb{R}^{N^2}$ is to map the low-quality tomograms reconstructed by IRM to the original object function model via regression. We employed the U-Net[4] architecture, as illustrated in Fig. 1, as the model for the image transformation task. The CNN is optimized using the Adam optimizer with a learning rate of 2×10^{-4}, and the loss function is the L1 norm error:

$$L(\boldsymbol{\theta}) = \left\|f_{\boldsymbol{\theta}}(\mathcal{R}(\boldsymbol{I})) - \boldsymbol{O}\right\|_1 \tag{3}$$

Here, $\boldsymbol{O} \in \mathbb{R}^{N^2}$ denotes the labels, $\boldsymbol{\theta}$ denotes the network parameters, and $\boldsymbol{I} \in \mathbb{R}^{nN}$ denotes the projections at various angles, $\mathcal{R}\{\cdot\}: \mathbb{R}^{nN} \rightarrow \mathbb{R}^{N^2}$ represents the IRM reconstruction. The L1 loss promotes sparse reconstruction, which helps suppress artifacts by preventing large weights from being assigned to outliers. For high-entropy datasets with high randomness, MAE not only drives the CNN to learn the underlying patterns of the inverse problem, but also helps avoid over-smoothed blurry regions.

In CNN inference, the initial input images were obtained by SART. When a tomogram was large, it was divided into smaller blocks using a sliding window. Artifacts were removed from each patch individually, and the results were then stitched together into a complete tomogram $\boldsymbol{O}_{\text{output}}$. However, this may produce visible seams at the edges of patches $\boldsymbol{O}_i$, which may interfere with image segmentation and atomic localization. To address this, Gaussian weight masks $\boldsymbol{G}_i$ were generated for each patch, and subjected to weighted summation and fusion, followed by brightness adjustment and normalization to obtain the final tomogram:

$$\boldsymbol{O}_{\text{output}} = \left(\sum_i \boldsymbol{G}_i \odot \boldsymbol{O}_i\right) \oslash \sum_i \boldsymbol{G}_i \tag{4}$$

Projection constraints are not used as a condition during either training or inference.

1.3 Experimental image preprocessing Image processing was performed using standard procedures. First, circular low-pass filtering was applied to remove high-frequency noise. The Anscombe transform and BM3D denoising[6] were then performed to remove Poisson–Gaussian noise[7]. For the denoised image, a mask slightly larger than the specimen was generated using the OTSU[8] algorithm. The contrast of the carbon film within the mask was estimated using the Telea algorithm[9] and then subtracted. Finally, tomviz[10] was used for alignment.

1.4 Evaluation metrics for nanoscale electron tomography For the nanoscale ET, we primarily employed the Fourier shell correlation to quantify reconstruction consistency. FSC is the core resolution metric for 3D reconstructions in electron microscopy[11, 12]. FSC procedure begins with two independent datasets, and two tomograms $\boldsymbol{x}, \boldsymbol{y}$ were reconstructed separately from the two datasets. Fourier transforms were then applied to these tomograms to obtain $\boldsymbol{X}, \boldsymbol{Y}$, which are subsequently divided into a series of concentric spherical shells S in 3D Fourier space based on spatial frequency k. The normalized cross-correlation coefficient was calculated within each shell, ultimately generating the FSC curve:

$$\text{FSC}(k) = \frac{\sum_{\boldsymbol{k}\in S} \boldsymbol{X}(\boldsymbol{k})\boldsymbol{Y}^*(\boldsymbol{k})}{\sqrt{\sum_{\boldsymbol{k}\in S} |\boldsymbol{X}(\boldsymbol{k})|^2}\sqrt{\sum_{\boldsymbol{k}\in S} |\boldsymbol{Y}(\boldsymbol{k})|^2}} \tag{5}$$

The above formula represents summing all points $\boldsymbol{k}$ within the spatial-frequency shell S at a given radius k. The FSC value ranges from 0 to 1, with values closer to 1 indicating higher similarity. In ET, criterion values between 0.143 and 0.5 can be used. Here, we adopt the relatively strict threshold of 0.5. Consequently, the actual resolution may be higher than the value estimated by the 0.5 criterion.

To avoid bias from a single metric in Fourier space, we further considered the local resolution (LR) in real space. LR is widely used for voxel-wise resolution evaluation in 3D reconstruction of cryo-EM[13, 14]. The LR of any voxel in the tomogram is defined as the minimum wavelength λ at which a local sine curve can be detected. LR approximates the local 3D characteristics at wavelength λ using a second-order Hermite polynomial with Gaussian windows and

conducts a likelihood ratio hypothesis test:

$$LRS = \frac{1}{2\sigma^2}\mathbf{S}^{\mathrm{T}}(\mathbf{\Gamma} - \mathbf{\Gamma}_0)\mathbf{S} \tag{6}$$

Where σ^2 is the noise variance estimated from the local voxel cube of the background region around the particle, $\mathbf{S}$ is the voxel neighborhood, and $\mathbf{\Gamma}, \mathbf{\Gamma}_0$ are covariance matrices under null and alternative hypotheses, respectively. The smallest λ detected at a given p-value (default 0.05) is defined as the local resolution of that voxel.

FSC and LR require independent reconstructions for cross-validation. In this study, the nanoporous carbon dataset collected at 2° intervals was divided into 3 independent tilt-series at 6° intervals. The micro-ET data (Co_3O_4 and MnO_2) and publicly available nanoscale ET data (Co_2P and PtCu) were divided into 2 independent tilt-series at 6° intervals. Their FSC and LR were calculated and averaged.

1.5 ADF-STEM image simulation The multi-slice simulation of AET was performed using the GPU-accelerated software package[5]. The 3D atomic model was placed inside the super-cell, which was then divided along the Z-axis into multiple 2.0 Å-thick slices, with the focal plane fixed at the center. The simulation parameters included −678 nm C_3 aberration, 361 μm C_5 aberration, 30 mrad convergence semi-angle, 60 and 200 mrad detector inner and outer angles, to simulate the ADF-STEM tilt-series. The sampling interval between the probe and the object was 0.04 Å, and 8 frozen phonons were simulated at each angle. The multi-slice image was then convolved with a Gaussian kernel of 100 pm to account for the electron probe and other incoherent effects.

1.6 Evaluation metrics for atomic electron tomography Unlike other fields of tomography, the truly meaningful results of AET are the atomic structures fitted from the tomograms. Therefore, the evaluation indicators are all based on the reconstructed atomic structures rather than the images themselves. For simulated AET tilt-series with known ground-truth, the search for common atomic pairs was first conducted between the reconstructed structure and the model structure, and then the RMSD of the common atomic pairs was calculated, defined as:

$$\mathrm{RMSD} = \sqrt{\frac{1}{m}\|\boldsymbol{r} - \boldsymbol{r}^*\|_2^2} \tag{7}$$

Here, $\boldsymbol{r}, \boldsymbol{r}^*$ denote the 3D coordinates of the *m* common atoms in the two sets of atomic structures. A lower RMSD and a higher common atomic ratio indicate a more accurate reconstruction. The common atomic ratio is defined as the number of common atomic pairs divided by the average total number of atoms in the two compared models[16, 17].

For AET experimental data with unknown ground-truth, most traditional AET methods use structures fitted from experimental tilt-series as surrogate models, and evaluate their reconstruction consistency via multi-slice simulated tilt-series[15]. In this study, the nanoparticle size is too large, and the multi-slice simulation requires an extremely fine sampling grid (~3 pm) to simulate high-angle scattering of 60–200 mrad, resulting in enormous computational cost. Therefore, we estimate the potential reconstruction error ε using two independent experimental datasets (odd and even

frames at each tilt-angle). Let $\bar{\boldsymbol{r}}$ denotes the unknown true structure, and the atomic structures obtained from two experiments can be written as $\boldsymbol{r}_1 = \bar{\boldsymbol{r}} + \boldsymbol{\varepsilon}_1$ and $\boldsymbol{r}_2 = \bar{\boldsymbol{r}} + \boldsymbol{\varepsilon}_2$. The deviations $\boldsymbol{\varepsilon}_1$ and $\boldsymbol{\varepsilon}_2$ can be regarded as two identical random variables, satisfying $\mathrm{Var}(\boldsymbol{r}_1 - \boldsymbol{r}_2) = \mathrm{Var}\big((\boldsymbol{r}_1 - \bar{\boldsymbol{r}}) - (\boldsymbol{r}_2 - \bar{\boldsymbol{r}})\big) = \mathrm{Var}(\boldsymbol{\varepsilon}_1) + \mathrm{Var}(\boldsymbol{\varepsilon}_2) = 2\varepsilon^2$. Thus, the RMSD between the reconstructed structure and the unknown true structure can be estimated by:

$$\mathrm{RMSD} = \varepsilon = \sqrt{\frac{1}{2m}\|\boldsymbol{r}_1 - \boldsymbol{r}_2\|_2^2} \tag{8}$$

Cross-validation has been used for crystal nucleation studies[16] and low-dose reconstruction of AET[33].

In addition, to evaluate the projection consistency of the reconstructed structure, the computed projection of the reconstructed structure was compared with the experimental projection. The normalized L1 norm errors (R_1-factor error and R_F-factor error) of the real-space projected images and reciprocal-space Fourier amplitudes are used as quantitative metrics[15-17, 34]:

$$R_1(\theta) = \frac{\left\|\boldsymbol{I}_{\mathrm{comp}}(\theta) - \boldsymbol{I}_{\mathrm{exp}}(\theta)\right\|_1}{\left\|\boldsymbol{I}_{\mathrm{exp}}(\theta)\right\|_1}, R_F(\theta) = \frac{\left\| \boldsymbol{F}_{\mathrm{comp}}(\theta) - \boldsymbol{F}_{\mathrm{exp}}(\theta) \right\|_1}{\left\| \boldsymbol{F}_{\mathrm{exp}}(\theta) \right\|_1} \tag{9}$$

Here, $\boldsymbol{F}_{\mathrm{comp}}$ and $\boldsymbol{F}_{\mathrm{exp}}$ represent the Fourier amplitudes of the computed projections $\boldsymbol{I}_{\mathrm{comp}}$ and experimental projections $\boldsymbol{I}_{\mathrm{exp}}$, respectively. In practice, the 3D object function $O(x, y, z)$ corresponding to the atomic structure $\{\boldsymbol{r}_i\}_{i=1}^{m}$ is first computed using the following formula:

$$O(x, y, z) = \sum_{i=1}^{m} H_i e^{-\frac{\pi^2}{B'}\|r\|^2} \otimes V_i(\boldsymbol{r} - \boldsymbol{r}_i) \tag{10}$$

Here, the H_i-factor is a scale factor to account for the influence of factors such as the collection angle on the relative atomic intensity. The B'-factor is to account for the influence of electron probe source size, spherical aberration, and thermal vibration; V_i is the atomic potential of atom i, derived from the electron scattering factor. Then, its computed projections were calculated using the corresponding forward model. The Adam optimizer is used for 200 iterations to optimize the H and B'-factor until the loss (R_1-factor error) converges, and the resulting error reflects a lower bound on projection consistency. All structures follow the same procedure for error evaluation.

Currently, a well-performed AET reconstruction for a small nanoparticle thinner than 8 nm usually has an average R_1-factor error of ~10%[15, 16, 18, 19, 21, 23, 25, 28]. However, as the number of atoms increases, the projection consistency error at the same precision will naturally decrease. For large-sized nanoparticles with more than 5×10^4 atoms, we believe that the R_1-factor error should be lower than 10% to be more reasonable.

Finally, to examine the structural changes of the specimens before and after AET, we also checked the consistency of the 0° projections before and after the experiment (Fig. S12).

1.7 Atom tracing After obtaining the atomic tomogram, atomic tracing was conducted using polynomial fitting and unbiased clustering methods commonly employed in AET[19]. First, the atomic tomogram was interpolated to generate a finer mesh. All local maxima were identified through breadth-first search, and potential atomic positions were extracted from the local volumes using a 3D fourth-order polynomial fitting method. The 3D tomograms were normalized to the range of 0–255, with a peak intensity threshold of 40 and a minimum spacing of 1.6 Å. The local volume for coordinate fitting was set to 8×8×8 voxels, with remaining parameters maintained at their default values. Finally, non-atomic peaks in the potential atom list were removed using k-means clustering.

1.8 Refinement of atomic coordinates To correct the potential deviations from imaging physics introduced by 2D approximation of 3D PSF, further refinement is required after obtaining the atomic structure. Inspired by established methods in regular AET literature[15, 18, 20], the atomic structure is refined using gradient descent to iteratively minimize the error $\left\|\boldsymbol{I}_{\text{comp}} - \boldsymbol{I}_{\text{exp}}\right\|_2^2$ between the experimental and computed projections of the atomic structure. First, the 3D object function $O(x, y, z)$ corresponding to the atomic structure $\{\boldsymbol{r}_i\}_{i=1}^{m}$ is computed using the Eq. (10), then, its computed projections were calculated using the corresponding forward model. To prevent over-fitting, the loss is modified as:

$$L = \alpha\left\|\boldsymbol{I}_{\text{comp}} - \boldsymbol{I}_{\text{exp}}\right\|_1 + \beta\left\|\boldsymbol{F}_{\text{comp}} - \boldsymbol{F}_{\text{exp}}\right\|_1 \tag{11}$$

The coefficients $\alpha = 1/\left\|\boldsymbol{I}_{\text{exp}}\right\|_1$ and $\beta = 1/\left\|\boldsymbol{F}_{\text{exp}}\right\|_1$ are used for normalization to ensure that the error value is independent of the image size. The first term in Eq. S(11) measures the overall deviation between the calculated and experimental projections in real space, ensuring projection consistency of the atomic structure. The second term serves as a regularization term, constructed from Fourier amplitudes to quantify the difference between the computed and experimental projections in the frequency domain, helping to preserve image edges and textures. All reconstructed atomic structures were optimized for 100 iterations using automatic differentiation combined with the Adam optimizer, stopping when the error was no longer decreasing. For AET of thick samples, the CNN outputs show little improvement before and after iterative refinement (Fig. S19), because the CNN has effectively captured the defocus inverse problem. Hence, this step can be omitted when computational resources are limited.

1.9 Calculation of the Shannon entropy To calculate the information entropy of different datasets, we randomly selected 10,000 images from the training sets of EM-CNN and ImgNet-CNN. The empirical distribution $\widehat{p}(\cdot)$ of an image $\boldsymbol{x} \in \mathbb{R}^{N_x N_y}$ can be approximated by the frequency of pixel values, from which the entropy $H(\boldsymbol{x})$ of each image can be computed:

$$H(\boldsymbol{x}) = -\sum_{k=1}^{K} \widehat{p}(x_k)\log_2\widehat{p}(x_k)\,,\ \text{with}\ \widehat{p}(x_k) = \frac{\sum_{i,j}\mathbb{1}\left\{[\boldsymbol{x}]_{i,j} = x_k\right\}}{N_x \times N_y} \tag{12}$$

The entropy of the tomograms was calculated by randomly selecting 10,000 2D tomograms from the public ET data reported in references[3, 15-31]. These tomograms cover a wide range of real materials, ranging from the atomic-scale to nanoscale, and thereby reflecting the complexity of general ET tasks. For entropy calculation, all images were converted into 256×256-pixel, 8-bit images.

1.10 Maximal Information Coefficient analysis CNNs for image transformation tasks usually have an encoder-decoder structure, where the encoder is responsible for extracting multi-scale features, the bridge layer exhibits a highly abstract characteristic, and the decoder is responsible for gradually recovering spatial details. Neural networks with strong generalization capabilities do not retain all the details of the training set. Instead, during the encoding process, they compress the redundant and dataset-specific information while retaining the information that is useful for output reconstruction. To quantitatively evaluate the information redundancy and independence among feature channels within each layer during the encoding-decoding process, we employ the Maximal Information Coefficient (MIC) to analyze feature correlations[32]. As a non-parametric statistic based on mutual information, MIC can effectively capture high-dimensional nonlinear dependencies. The mutual information between variables $\boldsymbol{x}$ and $\boldsymbol{z}$ can be understood as the decrease of the uncertainty in $\boldsymbol{x}$ given $\boldsymbol{z}$:

$$I(\boldsymbol{x}, \boldsymbol{z}) = H(\boldsymbol{x}) - H(\boldsymbol{x}|\boldsymbol{z}) \tag{13}$$

Where $H(\boldsymbol{x})$ is the Shannon entropy, and $H(\boldsymbol{x}|\boldsymbol{z})$ is the conditional entropy. To investigate inter-channel dependencies within the network, we registered forward hooks on selected convolutional blocks (e.g., residual and bridge layers) to capture activation maps during inference. Each activation tensor $\boldsymbol{F} \in \mathbb{R}^{C\times H\times W}$ within the same layer was reshaped into channel-wise vectors $\boldsymbol{f}_i \in \mathbb{R}^{H\times W}$ by flattening spatial dimensions. To ensure comparability, vectors were normalized using min–max scaling. For any channel pair, MIC is defined as:

$$MIC(\boldsymbol{f}_i, \boldsymbol{f}_j) = \max_{G_x, G_y} \frac{I_{G_x,G_y}(\boldsymbol{f}_i, \boldsymbol{f}_j)}{\log\left(\min\{|G_x|, |G_y|\}\right)} \tag{14}$$

Here, I_{G_x,G_y} denotes the empirical mutual information under a 2D grid partition (G_x, G_y), estimated based on histograms[32]. Within each layer, the MIC between all channel pairs is calculated to form a symmetric matrix $\boldsymbol{M} \in \mathrm{R}^{C\times C}$, and the mean of its off-diagonal elements is taken as the average correlation metric of that layer. In order to avoid the influence of the data itself, the input and output layers of the CNN do not participate in the statistics.

1.11 Frequency analysis of the decoder To investigate how different training datasets influence the spectral characteristics of priors, we performed frequency analysis of all convolutional filters in the decoder. We randomly selected 20,000 convolutional kernels (total number of kernels in the whole CNN model: 5,226,624). Each 3×3 2D convolutional kernel $\boldsymbol{\kappa}$ was transformed into the frequency domain using a zero-padded 2D fast Fourier transform, and its amplitude spectrum was calculated on a logarithmic scale:

$$M = \log(1 + |\mathcal{F}\{\boldsymbol{\kappa}\}|) \tag{15}$$

Then, using the center of $\boldsymbol{M}$ as the origin and 37.5% of the Nyquist radius as the boundary, the circle was divided into low-frequency (inner) and high-frequency (outer) regions. The amplitudes within each region were then summed, the ratio between the two indicates whether the learned kernels tend to capture smooth global structures (low-frequency dominant) or fine local details (high-frequency dominant).

1.12 Volumetric strain and ORR activity calculation First, an atom nearest to the mean position of the measured 3D atomic model was chosen as the origin of an FCC lattice. Using an initial FCC lattice constant, nearest-neighbor lattice sites were computed; if an experimental atom lay within 0.25× the nearest-neighbor distance of a site, it was assigned to that site. The nearest-neighbor search was iterated over newly assigned sites until no further assignments occurred. Next, the FCC lattice vectors were fitted to all assigned atoms by optimizing translation, rotation, and the lattice constant to minimize the positional error between measured atoms and lattice sites.

For each assigned Pt atom, a local neighbor subset was selected. With the atom set as the origin, local nearest-neighbor lattice sites were calculated based on the globally fitted lattice constant. If an experimental atom was found within 25% of the nearest-neighbor distance of a given site, it was assigned to that site. A local FCC lattice was then fitted, and the fitted lattice constant was taken as that atom's local lattice constant.

Surface atoms were classified into {111}, {100},{110} facets, and for each surface atom, the local volumetric strain $\varepsilon = (a_{loc} - a_{ref})/a_{ref}$ was computed, where a_{ref} is the bulk Pt lattice constant. ORR activity was computed according to the $[\varepsilon - \Delta E_{OH}]$ and $[\Delta E_{OH} - \ln(j/j_{\mathrm{Pt},(111)})]$ correlation obtained by DFT calculations[21, 35] (Fig. S27).

Supplementary Information Section 2: Baseline Methods for Comparison

2.1 nanoscale electron tomography For nanoscale electron tomography, we mainly compare the ImgNet-CNN, which is trained with random natural images, and the Tomo-CNN, which is trained with simulated tomograms, with the EM-CNN as the internal baseline. Compared with traditional filtered back-projection (FBP) and iterative reconstruction algorithms, such as Simultaneous Algebraic Reconstruction Technique (SART), Real-space Iterative Reconstruction (RESIRE), and Total Variation Minimization (TVM), the CNN models can all achieve certain improvements, but the EM-CNN always has the optimal performance (Fig. 2, Figs. S4-S8, Table. S1).

Furthermore, we also compared EM-CNN with the self-supervised electron tomography method IsoNet[36]. IsoNet is a specimen-specific self-supervised information completion method. It cuts subtomograms from the undersampled tomogram itself, rotates them to different orientations and trains them iteratively. It therefore requires sufficient learnable orientation/structure redundancy in the current tomogram, therefore, IsoNet is widely used for biological specimens. When IsoNet is directly applied to material specimens without structural redundancy, the tomogram is usually over-smoothed, resulting in the loss of high-frequency structural information; therefore, the 3D resolution is usually lower than that of the original reconstruction of traditional iterative methods, such as SART or RESIRE (Fig. S9). By contrast, our EM-CNN introduces large-scale random projections for pre-training. This not only overcomes the problem of limited generalization ability but also substantially improves the reconstruction resolution. Moreover, it has more advantages in practice. For example, for the Co_3O_4 nanosheet specimen (256^3 voxels), on a same RTX3090 GPU, EM-CNN only takes 20 seconds, while self-supervised IsoNet takes more than 10 hours.

2.2 Depth-dependent atomic electron tomography Although the concept of defocus-correction for AET of thick specimens has been reported[37-39], there is currently no open-source depth-dependent electron tomography reconstruction algorithm. Therefore, the CNN was mainly compared with linear-projection-based RESIRE and Generalized Fourier Iterative Reconstruction (GENFIRE), as shown in Table.1, Table. S2 and S3.

In addition, according to the description in Ref.[37], we implemented a depth-dependent iterative reconstruction program. Specifically, we iteratively minimized the error between the projection of the 3D tomogram $O \in \mathbb{R}^{N^3}$ convolved with the electron probe $P(z)$ at different depths and the experimental image $\boldsymbol{I}_{\text{exp}}$:

$$O=\arg\min_{\mathbb{R}^{N^3}} \frac{1}{2}\sum_{\theta} \left\| \sum_{z} P(z)\otimes O_{\theta}(x,y,z) - \boldsymbol{I}_{\text{exp},\theta} \right\|^2, \ P(z) = \left| \mathcal{F}_{xy}^{-1}\left\{ A(\boldsymbol{k}) e^{-i\pi\lambda\left(k_x^2+k_y^2\right)z} \right\} \right|^2 \quad (16)$$

We applied it to the large Pt nanoparticle, and it is observed that compared with the linear-projection-based iterative reconstruction method, the RMSD and common atomic ratio of depth-dependent RESIRE are improved (Fig. S26 a and b). However, the atomic features in its tomograms are still not obvious. This also indicates the necessity of introducing additional structural prior information during depth-dependent tomographic reconstruction. The essence of the defocus effect is the loss of high-frequency structural information. Therefore, correction of the physical model alone cannot completely overcome this problem.

Furthermore, the introduction of the defocus blur kernel will have a negative impact on the optimization process. Its

convergence rate decreased compared with the linear-projection-based RESIRE (Fig. S26c). The CNN method avoids the complex iterative inversion of depth-dependent tomography while effectively introducing both physical and structural prior information. It only requires constructing a dataset based on a deterministic defocus blur and projection process (this process does not have any hyperparameters that need to be tuned), and then using the CNN to perform direct inversion in an end-to-end paradigm, enabling efficient and accurate AET reconstruction of thick specimens.

Supplementary Information Section 3: Robustness Tests

3.1 Effect of the defocus blur kernels Ignoring the depth-dependent evolution of the PSF, we trained a CNN and applied it to AET reconstruction of thick samples (Fig. S18). The results indicate that the missing-wedge and insufficient data only account for part of the reconstruction error, while the influence of defocus cannot be ignored.

3.2 Validity of the 2D approximation In data-driven AET of thick specimens, a potential source of error in the CNN is the 2D approximation of 3D tomograms in the construction of the training set. This approximation ignores the PSF expansion and information blending along the tilt axis (y-direction). In practice, this approximation has not been found to introduce obvious errors. As shown in Figs. 4f,g, and Fig. S28b, the 2D tomograms of XZ and XY sections perpendicular to the tilt axis Y both present clear atomic profiles without stacking artifacts.

We also computed the forward projection of reconstructed structure using the 3D PSF. We then minimized the difference between the experimental and computed projections of the atomic structure, and compared atomic deviations and projection consistency errors before and after refinement. The results demonstrate that the 2D CNN has effectively learned the underlying inverse problem of depth-dependent PSF, the difference of consistency error in real space before and after 3D refinement was only 1% (Fig. S19).

Finally, the rationality of the approximation also depends on the symmetry of the optical system. The error is small for the rotational symmetry (spherical aberration and defocus-dominated lens), and the error will rise when there are anisotropic aberrations such as astigmatism. In the ET experiment, we carefully performed astigmatic correction before collecting the projected image at each tilt-angle.

3.3 Dynamical scattering For the thick-specimens containing heavy metal elements such as Pt, the electron beam may be disturbed by the atomic potential, thereby deviating from the theoretical PSF described in Eq. (2).

Near the tilt angles corresponding to the low-index zone axes, the consistency error between the computed projection and the experimental projection significantly increased (Fig. S15, Fig. S18), indicating that the current linear summation physical model cannot account for the channeling effect. It is recommended that during the experiment, the low-index crystal zone axes that may be encountered should be avoided by pre-rotation.

To assess the potential multiple scattering, we use the 13-nm Pt nanoparticle as an example, and take Eq. S(11) as the loss function, the object function model $O(\boldsymbol{r}, z)$ corresponding to the reconstructed atomic structure $\{\boldsymbol{r}_i\}_{i=1}^{m}$ and ADF-STEM image $\boldsymbol{I}_{\exp}$ as observed constraints, and the electron probe $P(\boldsymbol{r}, z)$ itself as a variable for iterative solution:

$$P(\boldsymbol{r}, z;\ \theta) = \arg\min \left\| \sum_{z} P(\boldsymbol{r}, z;\ \theta) \otimes O(\boldsymbol{r}, z;\ \theta) - \boldsymbol{I}_{\exp}(\theta) \right\| \quad (17)$$

We used both experimental and simulated data to fit the PSF disturbed by the Pt specimen. The first approach was to solve the PSF with the reconstructed atomic structure and the experimental image at θ= 25° as constraints (the maximum thickness of the specimen along the optical axis at this tilt angle). Another is computed the simulated ADF-STEM image at 25° tilt angle using the CNN-reconstructed structure via multi-slice simulation, and then solve the PSF

using the known ground-truth atomic model and the simulated image as constraints. The computed PSF indicates that no obvious deformation occurs on the electron probe (Fig. S20).

We further divide the experimental tilt-series into two independent sets of experimental data and estimate the uncertainties of atomic coordinates. We then compute the relationship between the atomic deviation and its average depth during the AET experiment. The average depth of the i-th atom is defined as:

$$d_i = \frac{1}{n}\sum_{\theta=1}^{n} z_{i\theta} \tag{18}$$

Here, n is the number of projections, $z_{i\theta}$ represents the distance between the i-th atom and focal plane in the θ-th tilt-angle. As shown in Fig. S20d, the accuracy gain is more pronounced for atoms whose average depth is above the focal plane, whereas the deviations of atoms with an average depth below the focal plane ($d_i < 0$) slightly increase due to the deformation of the electron probe caused by multiple scattering.

Therefore, for Pt particles beyond 13 nm, the improvement achieved by the CNN in reconstruction accuracy of bottom-layer atoms will no longer be obvious. If the 2D CNN is applied to thicker Pt specimens, it is necessary to consider discarding the parts of the region where the average depth di is below the focal plane. Since multiple scattering depends on the specific sample, it is difficult to provide a unified thickness limit. However, cross-validation can provide an easily testable criterion for credibility in practice.

3.4 Training set size We evaluate the effect of the training set size on reconstruction performance in Fig. S21.

3.5 Electron dose The reconstruction results in the Figs.4,5 employ all the projection data, and the total electron dose (6.21×10^5 e/Å^2) is comparable to that reported for conventional AET, the experimental projection of each tilt angle is obtained by stacking 10 rapidly acquired images. In order to explore the influence of electron dose on the reconstruction performance, we split it into a series of subsets independent of each other with different doses, and estimate the errors by cross-validation. Even under a low dose condition of 0.62×10^5 e/Å^2, the CNN can achieve an RMSD less than 30 pm (Fig. S22).

3.6 Residual aberrations

The 2D approximation assumes rotational symmetry in the defocus kernels. Real-world microscopy often involves residual astigmatism or coma. To analysis the robustness of our CNN model, we add triple astigmatism and coma to the phase to simulate an electron probe, $P(z) = \left|\mathcal{F}^{-1}\{Ae^{-i\chi}\}\right|^2$, with residual aberrations:

$$\chi(k_x, k_y, z) = -\pi\lambda\left(k_x^2 + k_y^2\right)z + A_3\lambda^2\left(k_x^2 + k_y^2\right)^{\frac{3}{2}}\sin\left(3(\phi - \phi_{A3})\right) + \frac{2\pi}{3}B_2\lambda^2\left(k_x^2 + k_y^2\right)^{\frac{3}{2}}\sin(\phi - \phi_{B2}) \tag{19}$$

Where λ is the wavelength, k_x, k_y are the frequencies in Fourier space, z is the depth, A_3 is the triple astigmatism, B_2 is the coma, ϕ_{A3}, ϕ_{B2} are the aberration angles, and $\phi = \tan^{-1}\left(k_y/k_x\right)$ is the azimuth angle of the frequency point in

Fourier space. Since ϕ_{A3}, ϕ_{B2} only affect the direction of the aberration, we set them to 0 and generate a series of electron probes (Figs. S23a–c) at the usual magnitude of the residual aberration in spherical aberration electron microscopy. We then generate a series of simulated data using the Pt particle resolved in this work as the structural model, convolve the electron probe with the atomic potential of the nanoparticle and compute its projection, and then evaluate the reconstruction consistency. The CNN is robust to the residual aberrations (Figs.S23d–i).

Supplementary Information Section 4: Specimens Preparation

4.1 Nanoporous carbon The nanoporous carbon was purchased as commercial Ketjen Black EC300J carbon (specific surface area ~800 m^2/g, Lion Corporation, Japan).

4.2 Co_3O_4 nanosheets The Co_3O_4 nanosheets were synthesized by a liquid-precipitation route. Ultrathin $Co(OH)_2$ nanosheets were first prepared employing the cationic surfactant cetyltrimethylammonium bromide (CTAB) and the mild reducing agent $NaBH_4$. The resulting precipitate was collected by centrifugation, washed three times with acetone, and dried under vacuum at 60 °C to obtain $Co(OH)_2$ powder. Subsequent calcination in air at 400 °C for one hour induced a solid-state conversion of $Co(OH)_2$ into spinel Co_3O_4, while maintaining the original nanosheet morphology.

4.3 MnO_2 nanowires The α-MnO_2 nanowires were synthesized by the hydrothermal method. Dissolve 1.2 g of $KMnO_4$ and 0.5 g of $MnSO_4 \cdot H_2O$ in 60 mL of deionized water and ultrasonically treat for 5 minutes. Then transfer the liquid to the 100mL Teflon inner liner of the high-pressure autoclave. React in a hydrothermal oven at 140 ℃ for 12 hours. The solid-liquid mixture after the reaction was separated using a high-speed centrifuge, and then rinsed and centrifuged twice with ultrapure water and ethanol respectively. The solid was dried in a freeze dryer for 12 hours to obtain the powder sample. Finally, the freeze-dried powder is heat-treated at 400 ℃ for 2 hours.

4.4 Octahedral Pt nanoparticles The octahedral Pt nanoparticles were prepared via the organic-phase method. Using a condensation reflux setup with magnetic heating and stirring, ethylene glycol was added to a three-neck flask and heated in air at 110 °C for 1 h to remove water. Chloroplatinic acid (H_2PtCl_6) and polyvinylpyrrolidone (PVP, Mw 55,000) were dissolved separately in ethylene glycol at room temperature, with the molar ratio of H_2PtCl_6 to PVP set at 1:5. The two solutions were simultaneously added dropwise to the reaction flask over 90 s. The reaction mixture was heated under air at 110 °C for 1.5 h, observing a solution color change from golden-orange to greenish-yellow. The reaction mixture was further heated under N_2 for 30 min, turned brown, and became dark brown after an additional 1 h. After the reaction was completed, the mixture was cooled to room temperature, transferred to a centrifuge tube, mixed with ethanol and centrifuged. The resulting octahedral Pt nanoparticles were collected and dispersed in ethanol, then stored in a refrigerator. At last, 1 mL of the dispersed solution was diluted and deposited onto a copper mesh grid to prepare the electron microscopy sample. Prior to the AET experiment, the prepared sample grid was heated at 120 °C, cleaned with a plasma cleaner under Ar/H_2 atmosphere, and was finally subjected to a beam shower to remove surface carbon.

Figures

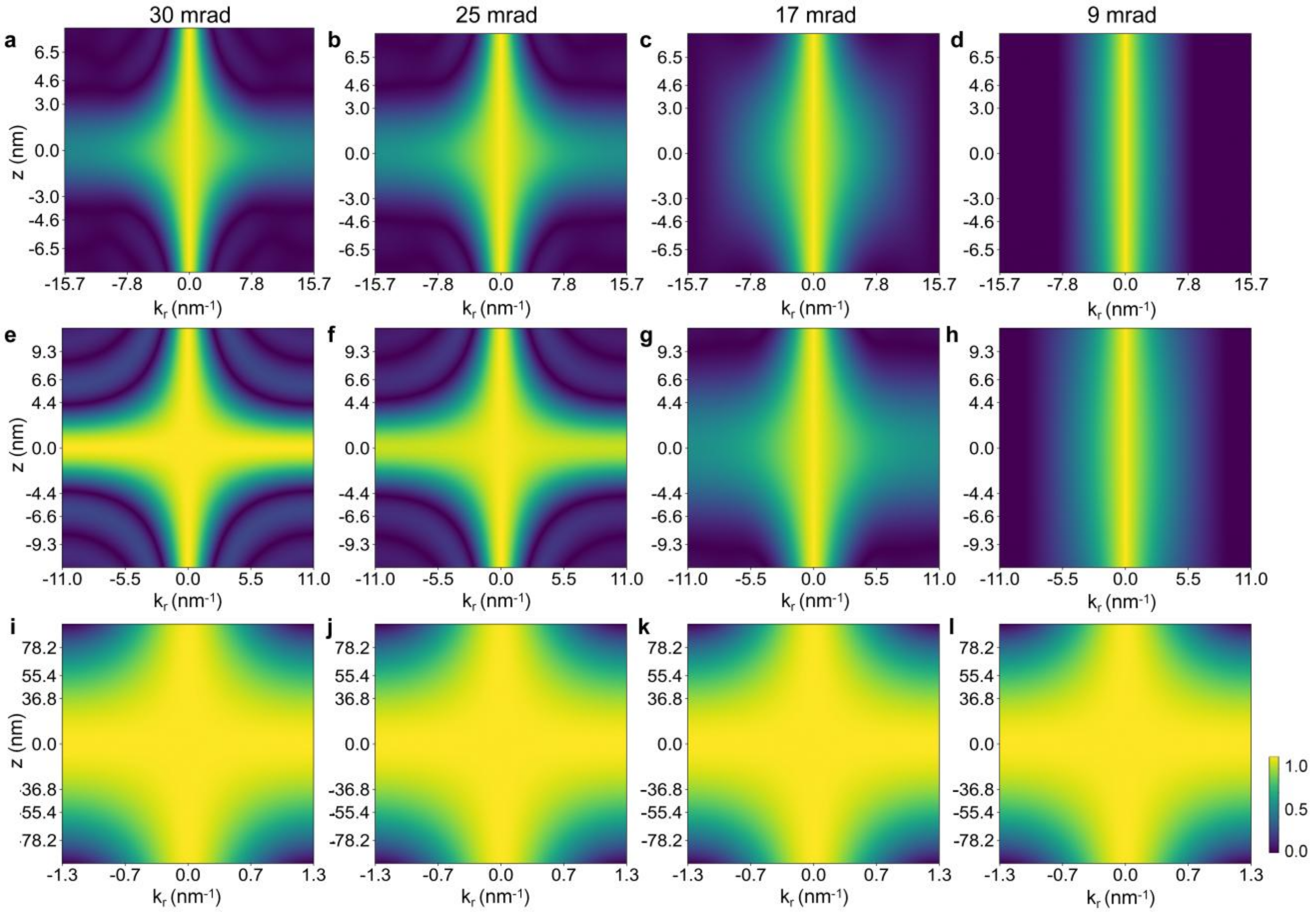

Fig. S1. Visualization of radial cross-sections $H(k_r, z)$ **of defocus-dominated 3D transfer function at different resolutions and convergence semi-angles. a–d,** Voxel size: 0.3185 Å (atomic scale resolution). **e–h,** Voxel size: 0.454 Å (atomic scale resolution). **i–l,** Voxel size: 3.8 Å (nanoscale resolution). The transfer function of the large-sized Pt particle is (a), the high-frequency structural information decays rapidly at ~3nm out of the focal plane and leads to a depth-dependent non-uniform blur.

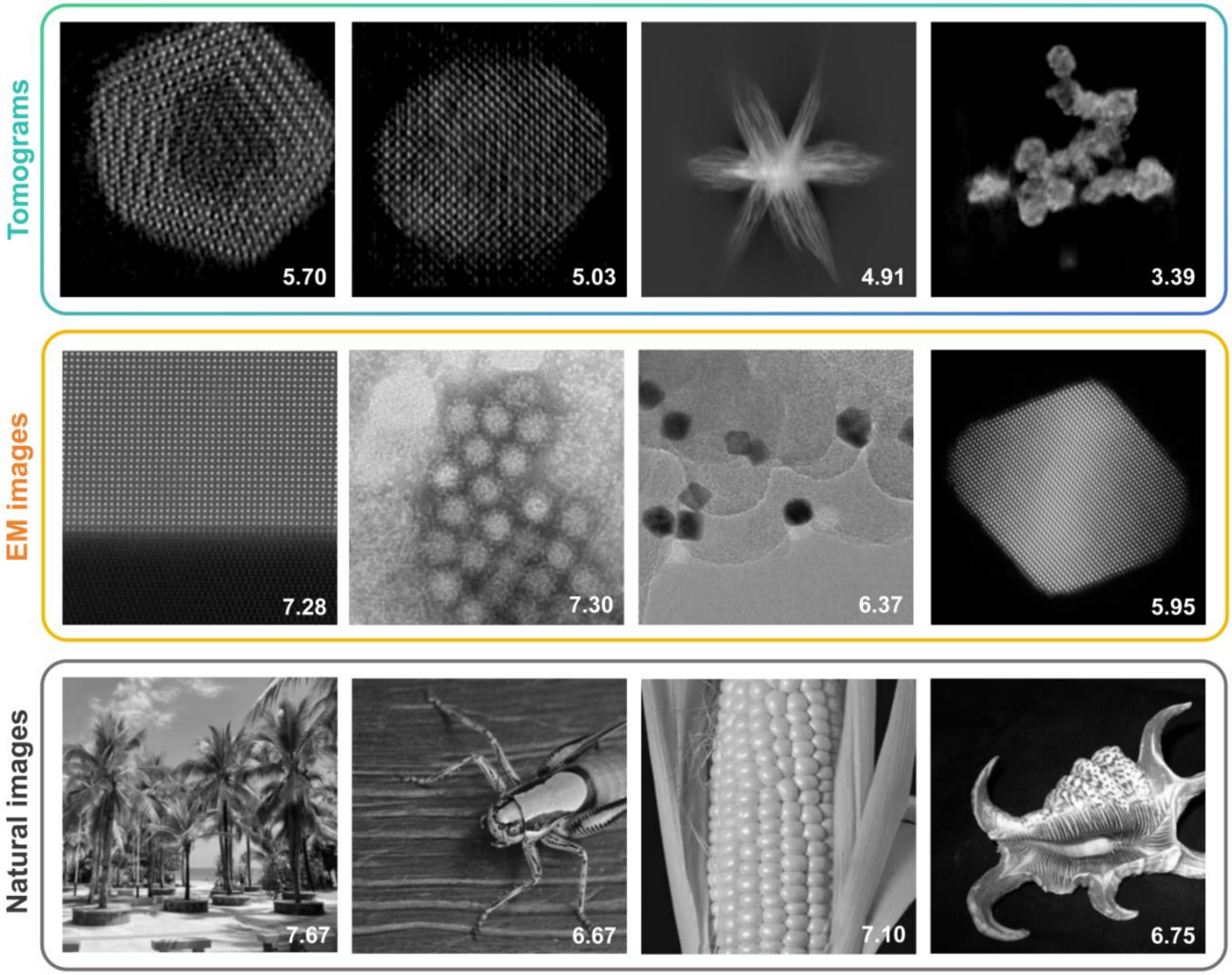

Fig. S2. Representative samples from different datasets and their entropies. Shown from top to bottom are the dataset of electron tomograms, electron microscopy images, and natural images. The information entropy of each is shown in the bottom right corner. Extensive and readily available random EM images, while providing content prior knowledge of the microscopic world, can drive neural networks to learn the fundamental principles of inverse problems, driven by their high-entropy characteristics, thereby achieving a better balance between accuracy and generalization.

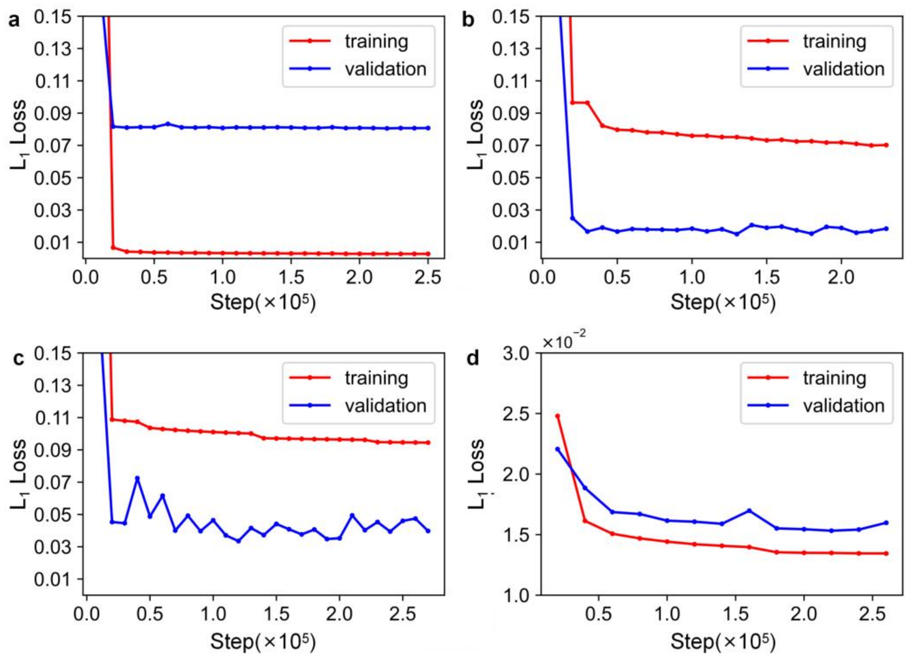

Fig. S3. Training curves of neural networks. a, Tomo-CNN. **b,** EM-CNN. **c,** ImgNet-CNN. Both the training and validation sets in (**a**) consist of low-entropy tomograms. When trained with artificially constructed atomic tomograms of nanoparticles, the model rapidly reduces the loss by memorizing and replicating features of the atomic potential, but converges to local minima. It shows high loss on validation sets composed of tomograms with different components, structures, and resolutions, and cannot be further optimized. In (**b**) and (**c**), the high-entropy random image training set enables the neural network to learn the underlying physics of the under-sampled tomographic inverse problem. As a result, the neural network exhibits lower validation loss on unseen low-entropy tomograms than on the training set. Because random EM images provide content priors, the validation loss is lower and the training process is more stable. **d,** Defocus corrected AET model. Both the training and test sets consist of high-entropy random microscopic images with similar complexity, showing a conventional training curve.

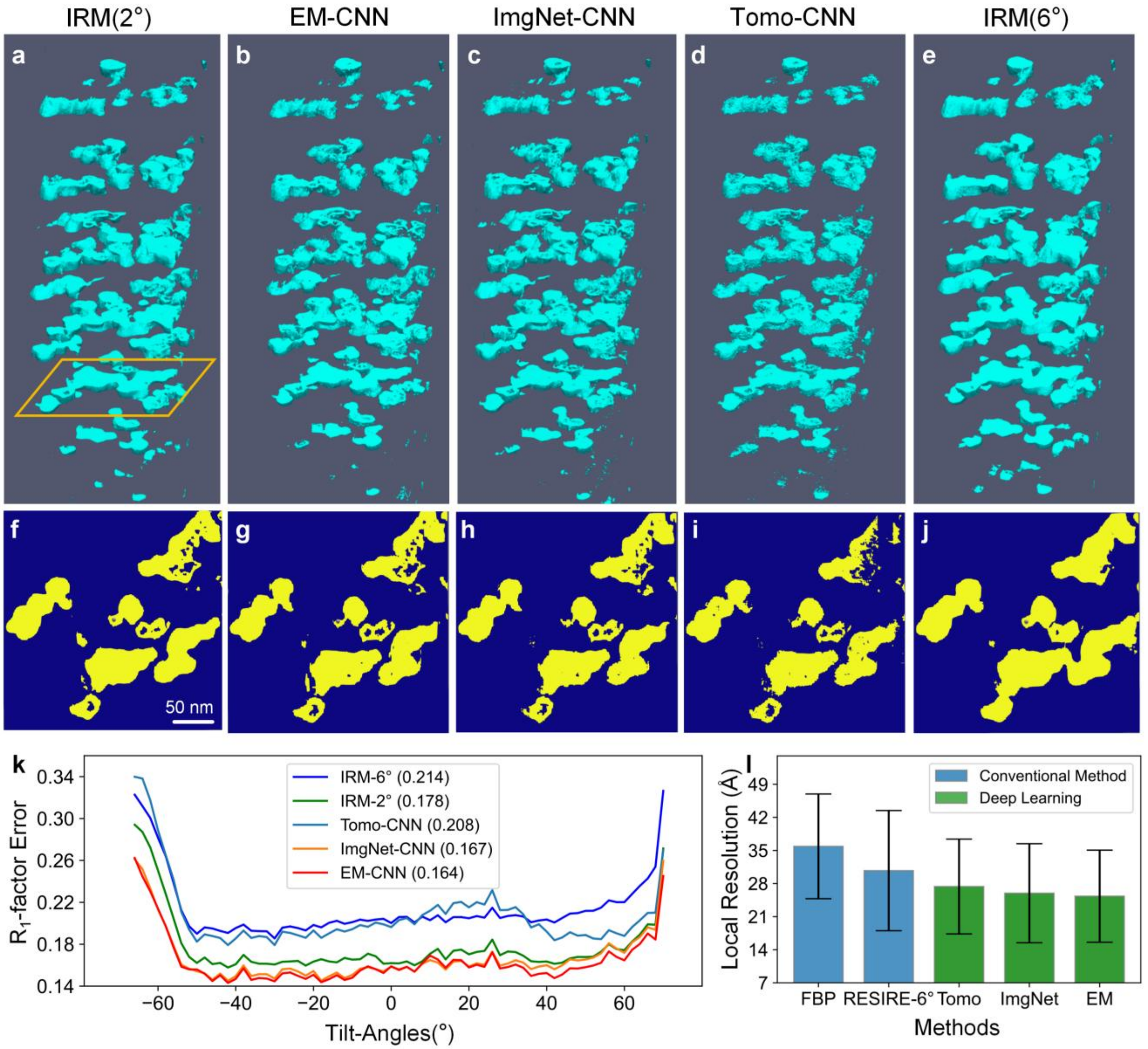

Fig. S4. Three-dimensional morphology and real-space consistency validation of EC300J nanoporous carbon material. **a–j,** 3D morphology structure models (**a**)–(**e**) of different reconstructed voxel images segmented by the OTSU algorithm and their 2D cross-sections (**f**)–(**j**) in the orange region of (**a**). **k,** The R_1-factor error between the simulated tilt-series computed based on the 3D segmentation results and the experimental tilt-series, with the average R_1-factor in the legend. **l,** Real space local resolution statistics for different reconstruction results obtained by cross-validation, with error bars indicating the standard deviation. The reconstruction results of EM-CNN still have the lowest projection consistency error and highest average resolution.

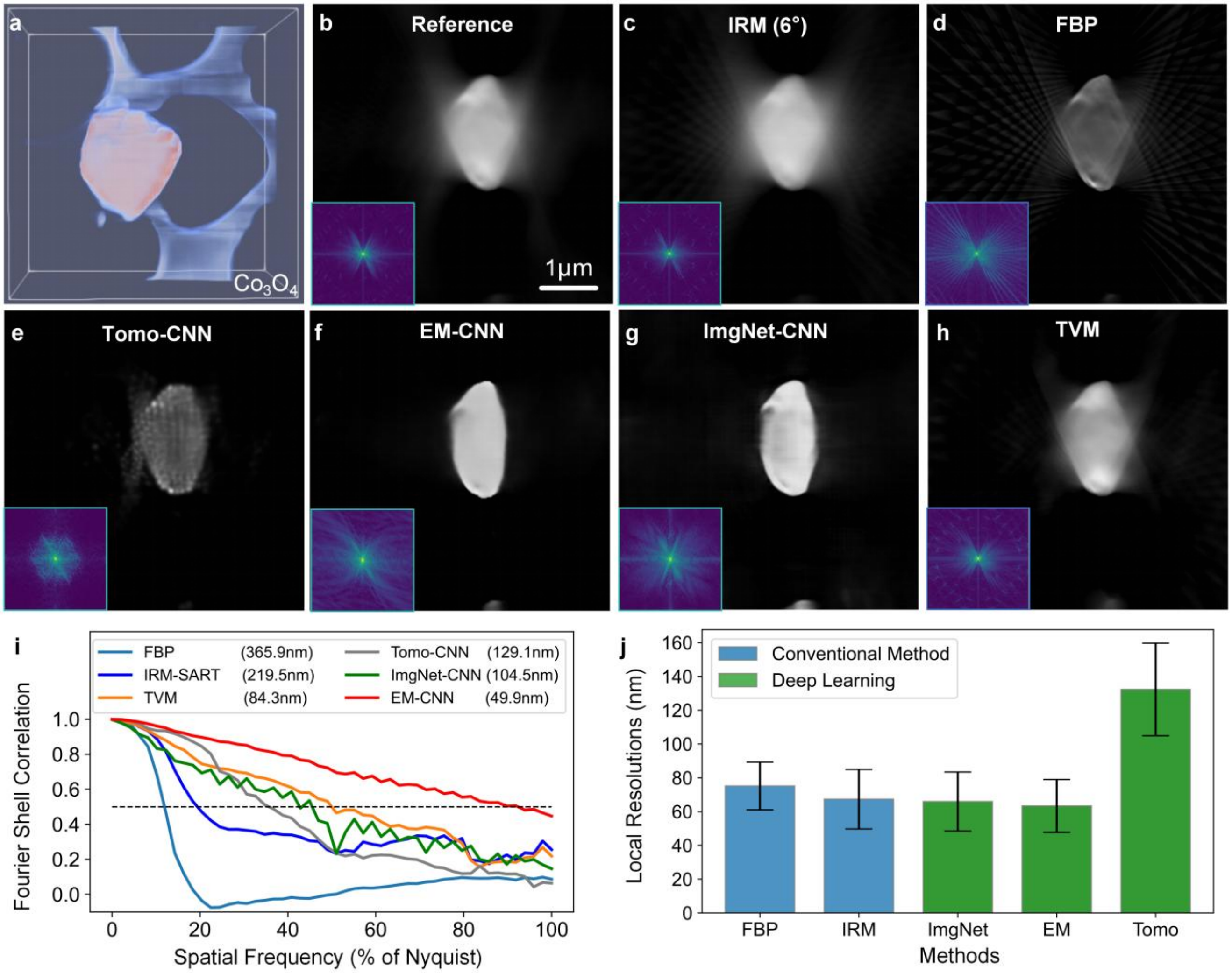

Fig. S5. Blind test of data-driven micro-ET reconstruction of Co_3O_4. a, 3D tomogram of Co_3O_4 material reconstructed by EM-CNN at 6° intervals (512^3 voxels, with a voxel size of 22.4 nm). **b–c**, IRM (SART) tomograms reconstructed from experimental tilt-series of 3° intervals (**b**) and 6° intervals (**c**). **d**, Tomograms reconstructed by the direct inverse transform method Filtered Back-Projection (FBP). **e–g**, Tomograms reconstructed by different CNNs. **h**, Tomograms reconstructed by the prior regularization method Total Variation Minimization (TVM). **i,** FSC curves for each method. **j,** Voxel-wise local resolution statistics for different reconstruction results obtained by cross-validation, with error bars indicating the standard deviation.

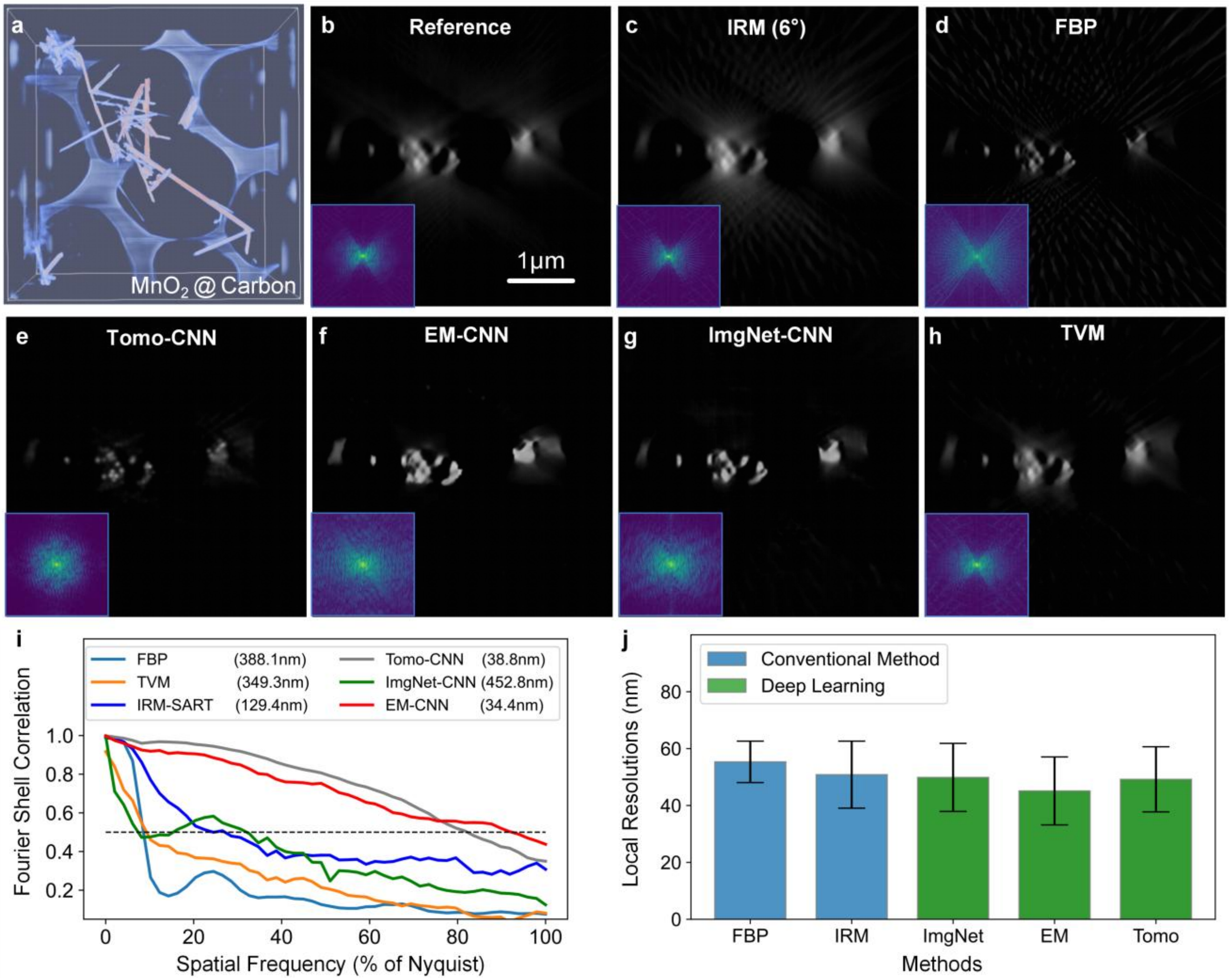

Fig. S6. Blind test of data-driven micro-ET reconstruction of a MnO_2 nanowire. a, 3D tomogram of MnO_2 material reconstructed by EM-CNN at 6° intervals (512^3 voxels, with a voxel size of 15.86 nm). **b–c**, IRM (SART) tomograms reconstructed from experimental tilt-series of 3° intervals (**b**) and 6° intervals (**c**). **d**, Tomograms reconstructed by the direct inverse transform method Filtered Back-Projection (FBP). **e–g**, Tomograms reconstructed by different CNNs. **h**, Tomograms reconstructed by the prior regularization method Total Variation Minimization (TVM). **i,** FSC curves of each method. **j,** Voxel-wise local resolution statistics for different reconstruction results obtained by cross-validation, with error bars indicating the standard deviation.

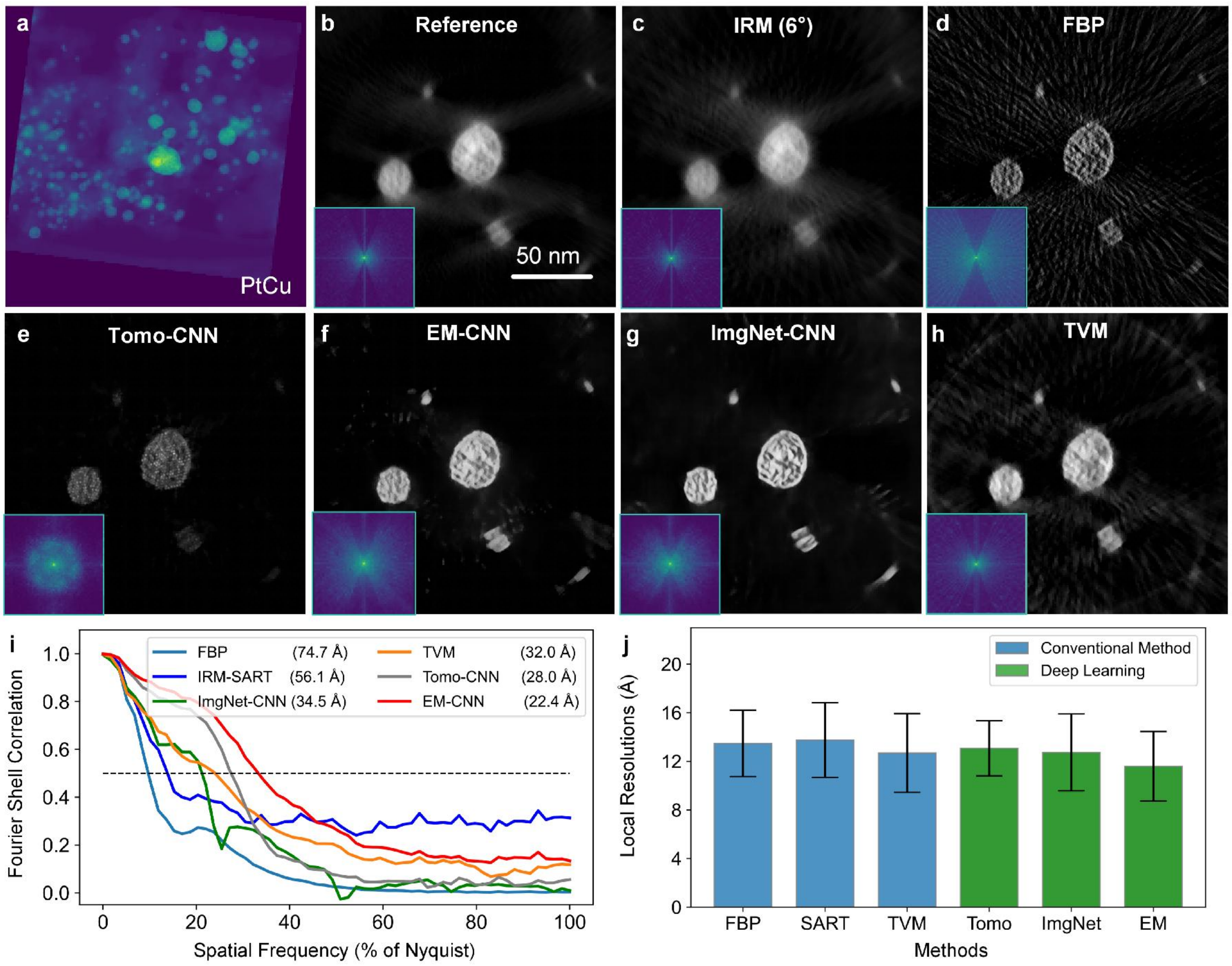

Fig. S7. Blind test of data-driven nano-ET reconstruction of a nanoporous PtCu catalyst from publicly available dataset[3]**. a,** An ADF-STEM projected image of PtCu catalyst, with a size of 1,145×1,145 and a pixel size of 3.8 Å. **b–c**, IRM (SART) tomograms reconstructed from experimental tilt-series of 3° intervals (**b**) and 6° intervals (**c**). **d**, Tomograms reconstructed by the direct inverse transform method Filtered Back-Projection (FBP). **e–g**, Tomograms reconstructed by different CNNs. **h**, Tomograms reconstructed by the prior regularization method Total Variation Minimization (TVM). **i,** FSC curves of each method. **j,** Voxel-wise local resolution statistics for different reconstruction results obtained by cross-validation, with error bars indicating the standard deviation.

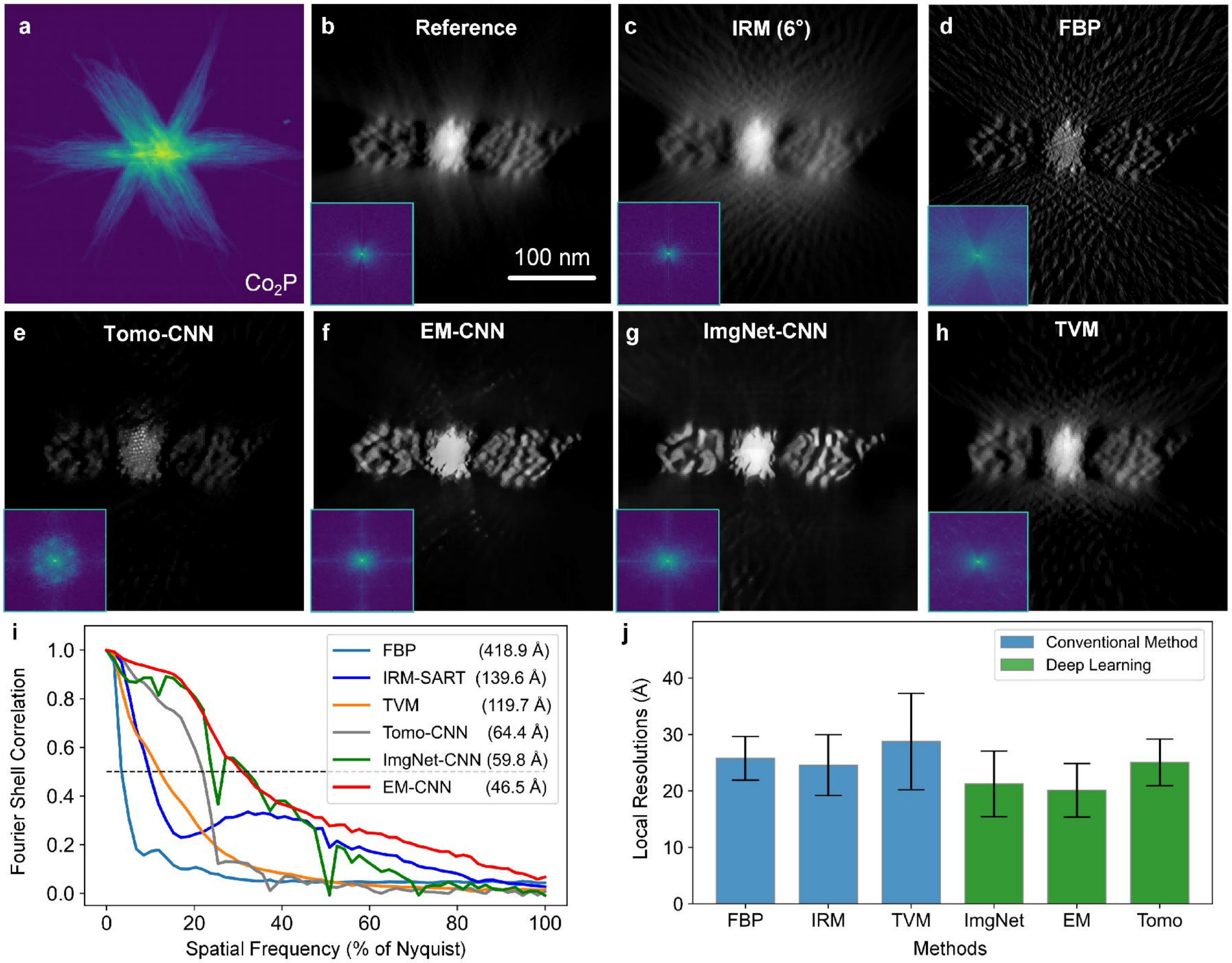

Fig. S8. Blind test of data-driven nano-ET reconstruction of a Co_2P nanocrystal from publicly available dataset[3]. **a,** An ADF-STEM projected image of Co_2P, with a size of 1,157×1,157 and a pixel size of 7.1 Å. **b–c**, IRM (SART) tomograms reconstructed from experimental tilt-series of 3° intervals (**b**) and 6° intervals (**c**). **d**, Tomograms reconstructed by the direct inverse transform method Filtered Back-Projection (FBP). **e–g**, Tomograms reconstructed by different CNNs. **h**, Tomograms reconstructed by the prior regularization method Total Variation Minimization (TVM). **i,** FSC curves of each method. **j,** Voxel-wise local resolution statistics for different reconstruction results obtained by cross-validation, with error bars indicating the standard deviation.

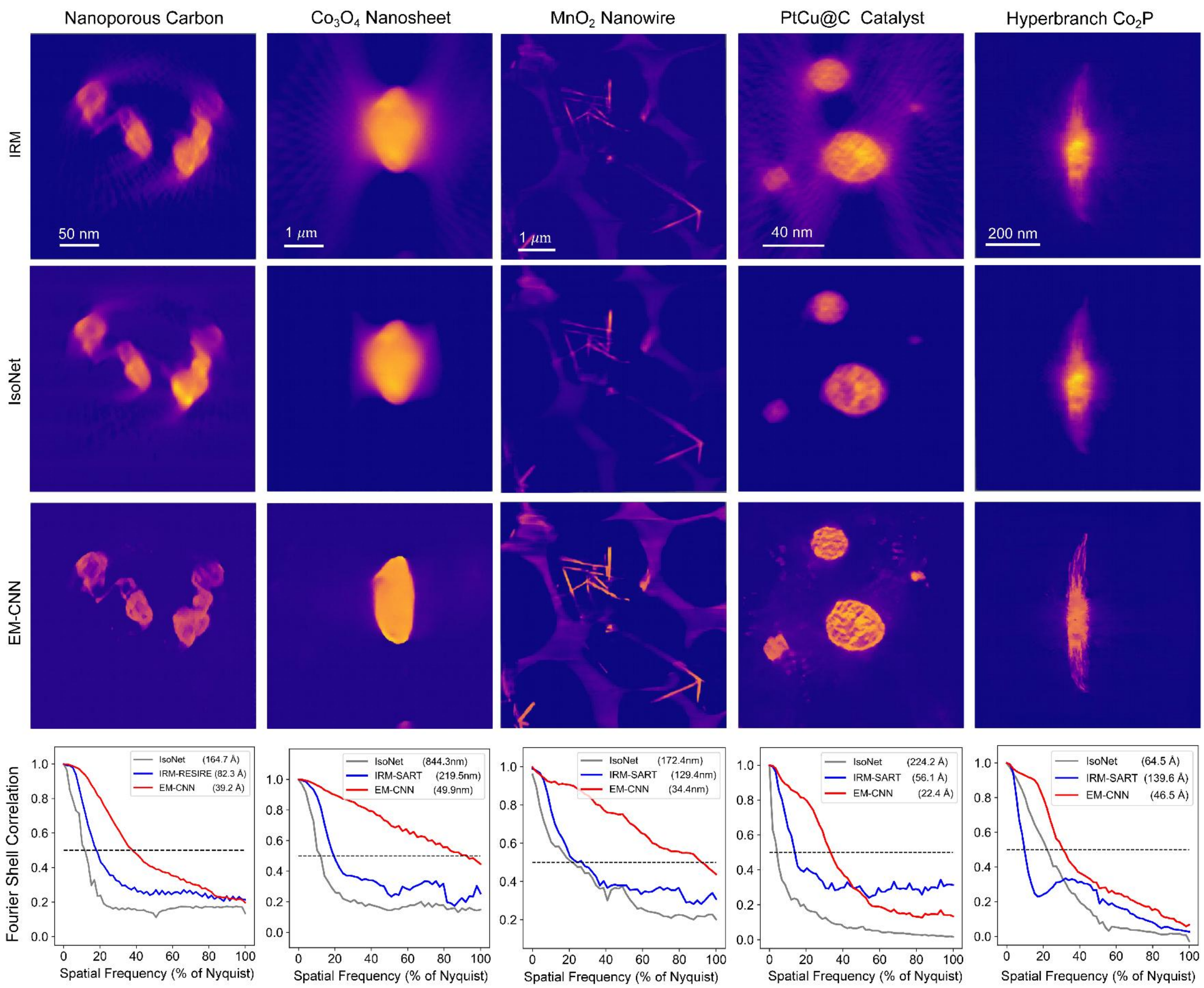

Fig. S9. Comparison of EM-CNN and self-supervised deep-learning method. From top to bottom: raw tomogram of traditional iterative reconstruction, tomogram processed by IsoNet[36], tomogram processed by EM-CNN (ours), Fourier shell correlation curve via cross-validation (the 3D resolution in the legend is estimated by the 0.5-criterion). EM-CNN is more suitable for nanoscale ET of materials specimens without structural redundancy.

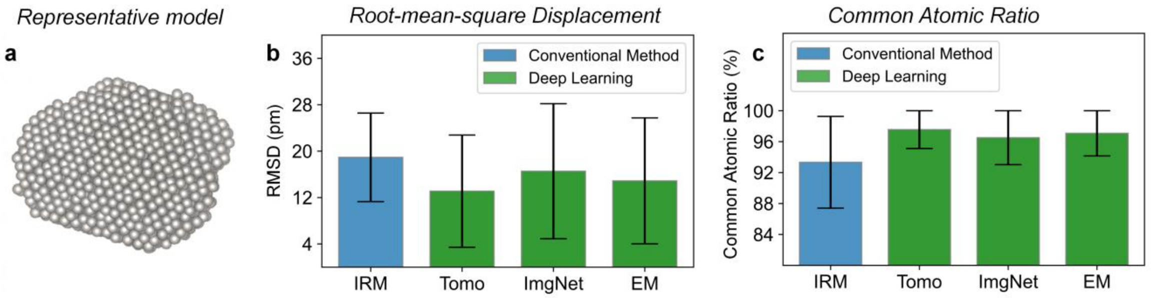

Fig. S10. Data-driven AET reconstruction tests of simulated nanoparticles. a, Representative random atomic structures. **b**, RMSDs before and after applying CNNs. **c**, Common atomic ratios before and after applying CNNs.

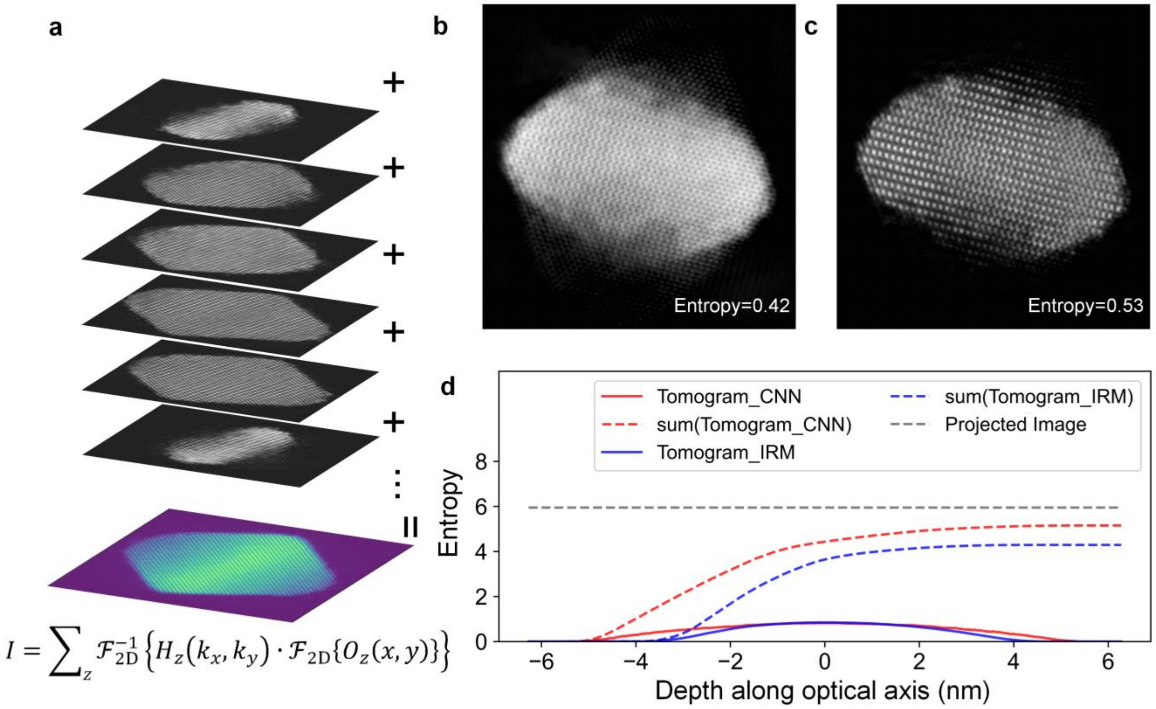

Fig. S11. Relationship between the entropy of tomograms and that of projected images. **a**, Relationship between tomograms and 0° projected image. **b–c**, Representative tomogram before (**b**) and after (**c**) CNN augmentation (with a defocus $\Delta f =-3.5$ nm), the entropy of the tomogram increases due to the lost information being recovered, especially in regions where the averaged depth is far from the focal plane. **d**, During the projection process, the information entropy increases as the tomograms are superimposed. As a result, the entropy value of random EM images is almost always larger than that of tomograms. *Tomogram_IRM* is the tomogram reconstructed by IRM, *Tomogram_CNN* is the tomogram augmented by the neural networks.

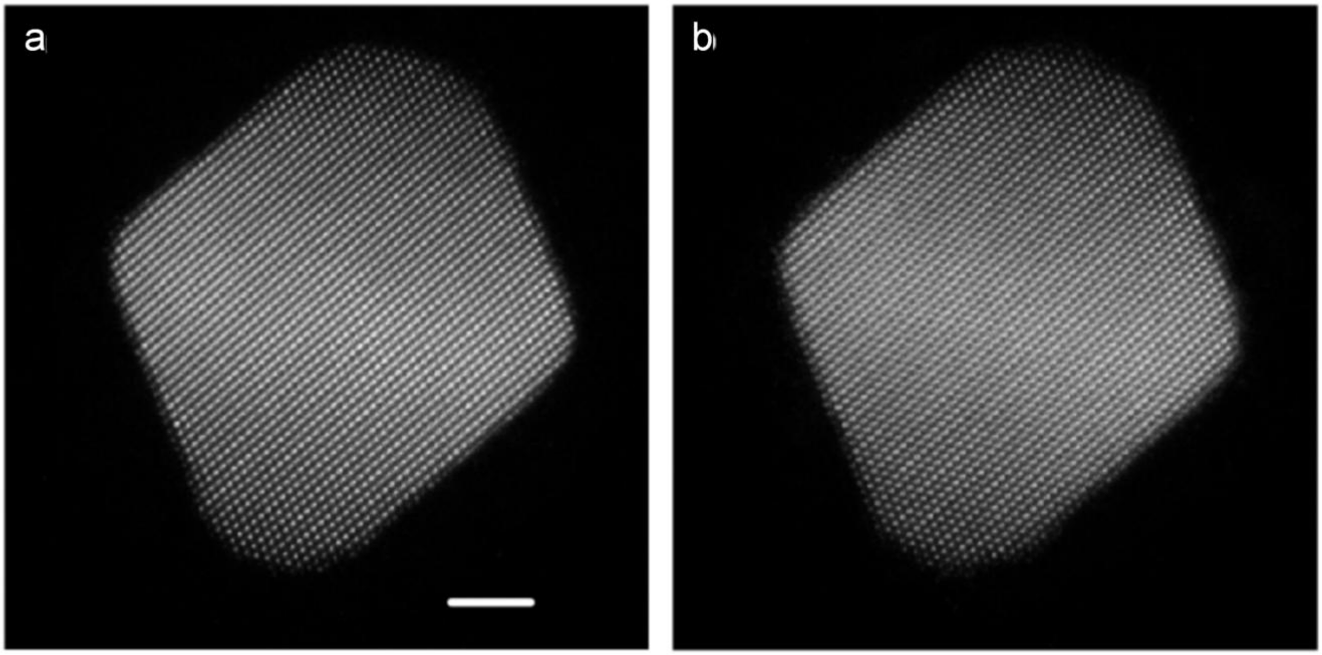

Fig. S12. Irradiation damage analysis. a–b, The 0° projections of the truncated octahedral Pt nanoparticle before (**a**) and after (**b**) the tomographic experiment. Scale bar, 2nm.

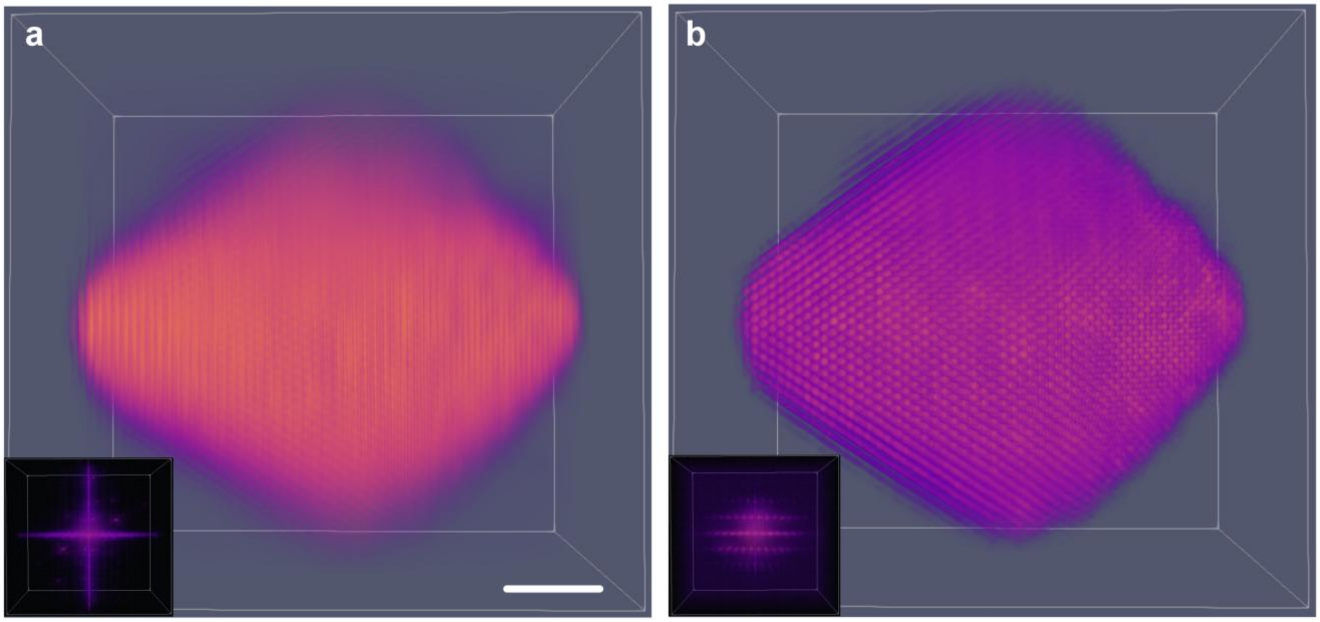

Fig. S13. Three-dimensional visualization of a 13-nm Pt nanoparticle. 3D rendering of the reconstructed volume, with its logarithmic Fourier amplitude shown in the lower left corner. **a**, The IRM result, and a blurred artifact is present in the area far from the focal plane. **b**, The CNN result presents a clear 3D lattice in both real space and Fourier space. Scale bar, 2 nm.

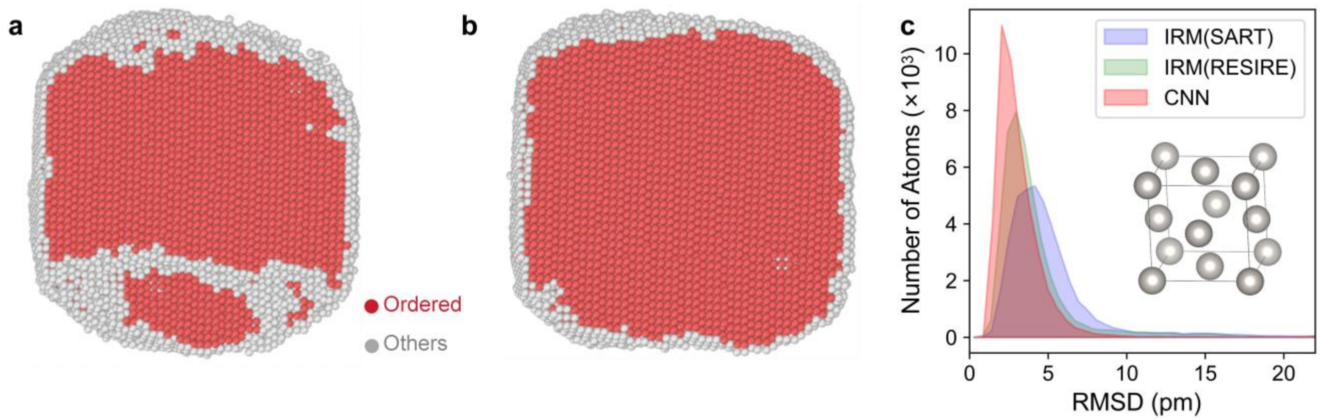

Fig. S14. Improvement visualization of a 13-nm Pt nanoparticle. a–b, FCC lattice matching[40] results of bulk atoms before **(a)** and after CNN augmentation **(b)**, respectively. **c,** Polyhedral template RMSD histogram of FCC atoms.

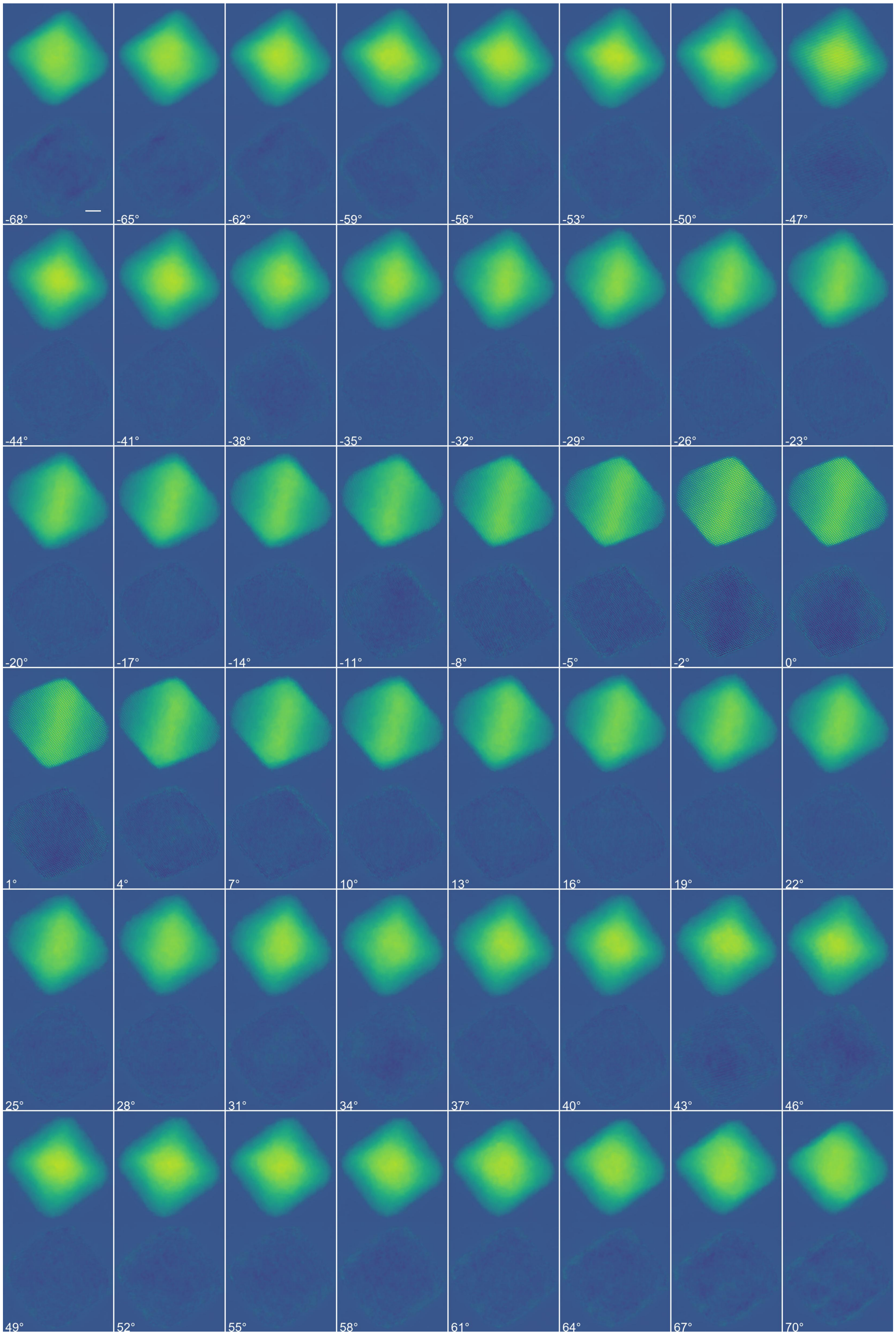

Fig. S15. Consistency of the truncated octahedral Pt nanoparticle reconstructed by CNN. The ADF-STEM experimental images at different tilt-angles (top) and the error images of the atomic structure (bottom). Scale, 2nm.

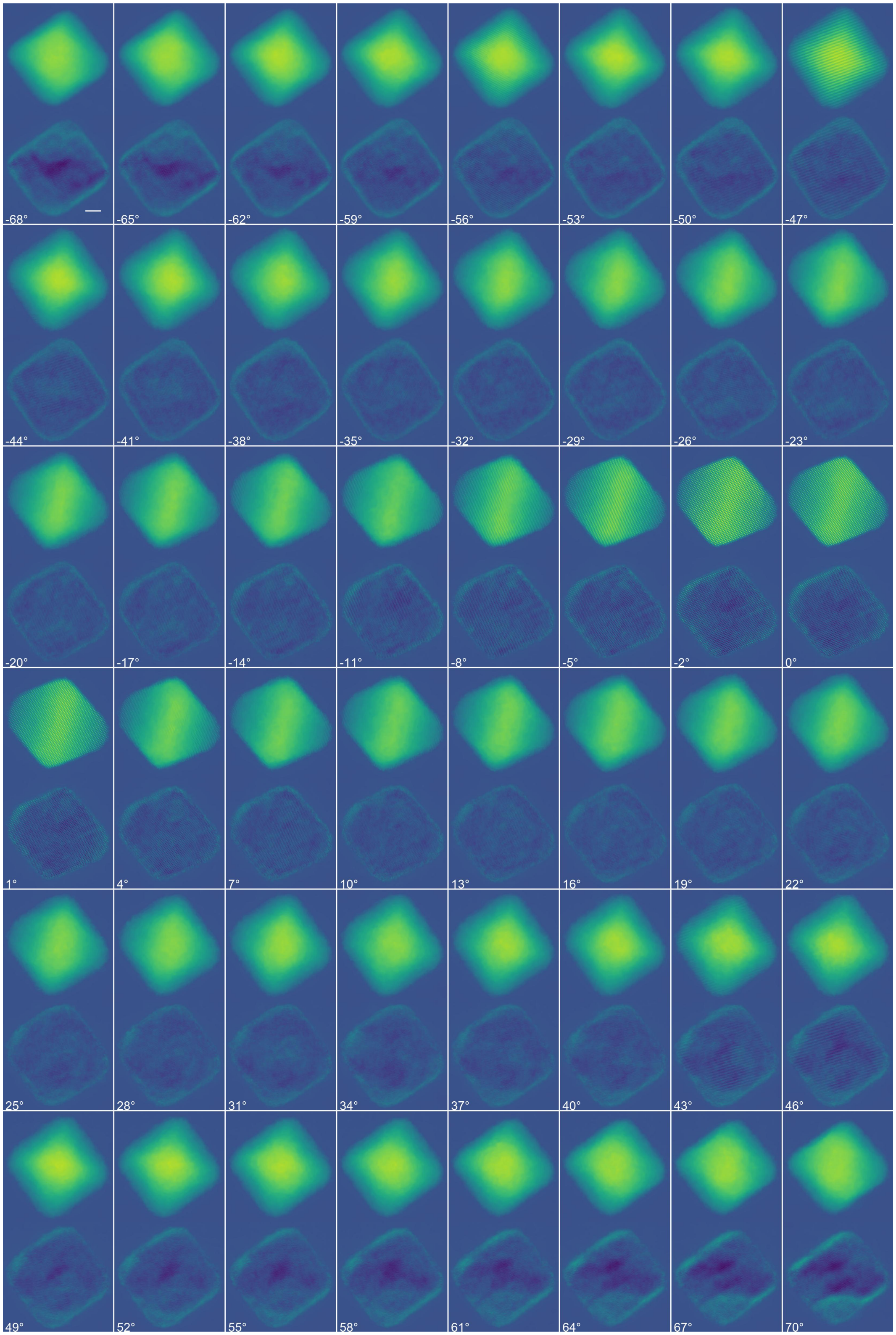

Fig. S16. Consistency of the truncated octahedral Pt nanoparticle reconstructed by IRM. The ADF-STEM experimental images at different tilt-angles (top) and the error images of the atomic structure (bottom). Scale, 2nm.

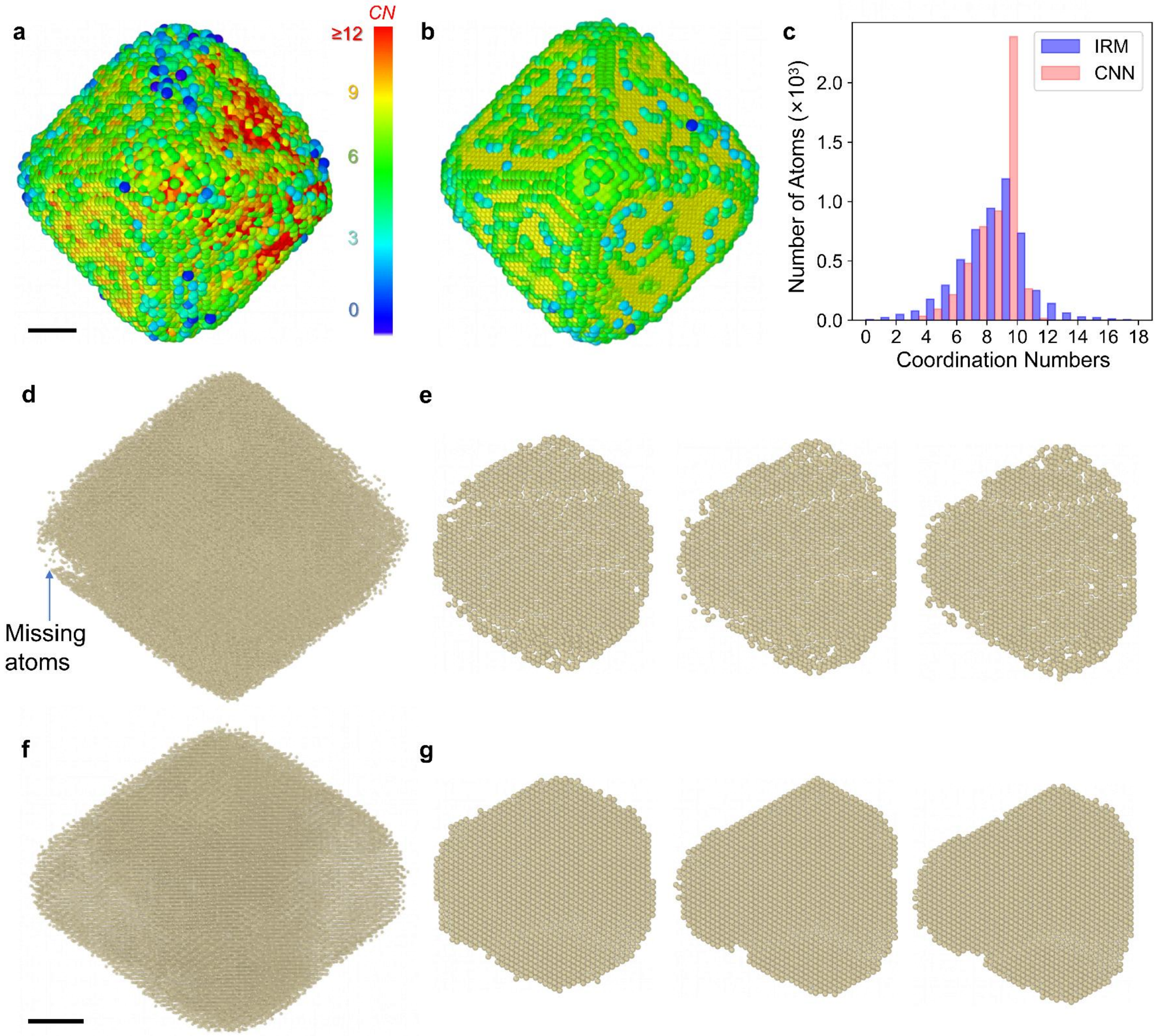

Fig. S17 Improvement visualization of the surface structure of a 13-nm Pt nanoparticle. a–b, Coordination numbers of the atomic structure before **(a)** and after CNN augmentation **(b)**, respectively. **c,** Statistics of surface atomic coordination numbers. Surface atoms were identified using the alpha-shape method of OVITO software, with a probe sphere radius of 2.7 Å and a smoothing level of 50. **d–e**, Atomic structure **(d)** and internal slices **(e)** reconstructed by IRM, where the blue and red circles indicate regions of structural disorder and atoms that have migrated beyond the surface, respectively. **f–g**, Atomic structure **(f)** and internal slices **(g)** reconstructed by CNN. Scale bar, 2 nm.

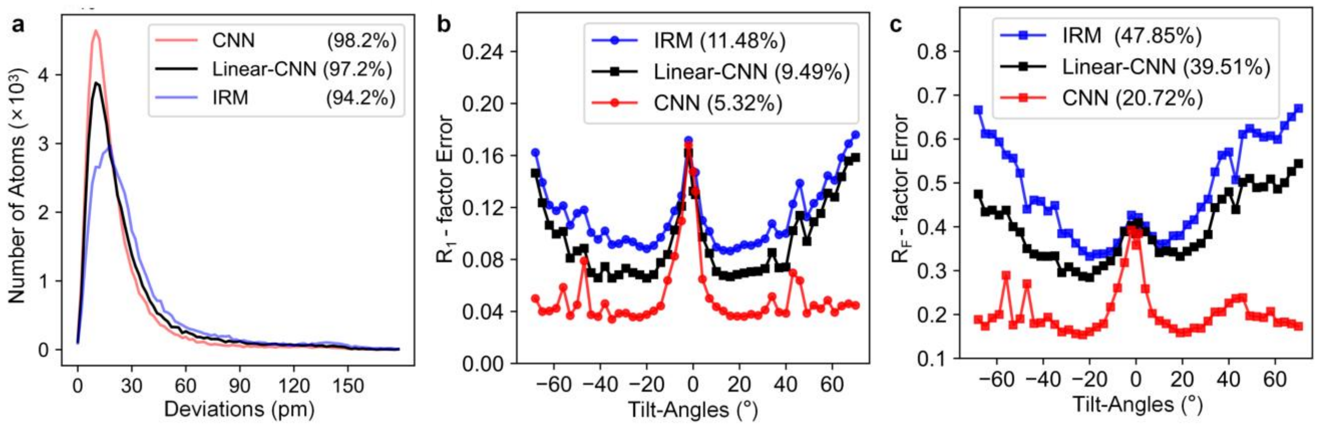

Fig. S18. Ablation study on the introducing the defocus blur kernel in 3D reconstruction of thick specimens. The training set of Linear-CNN ignores the depth-dependent evolution of the PSF and only addresses the under-sampled effect. **a,** Histogram of atomic deviations obtained by cross-validation with two independent experimental datasets, with the common atomic ratio given in parentheses. The RMSD of Linear-CNN is 27.5 pm, lying between that of IRM (32.6 pm) and CNN with defocus correction (22.6 pm). **b–c**, Real-space R_1-factor errors (**b**) and Fourier-space R_F-factor errors (**c**), with their average values in the legend.

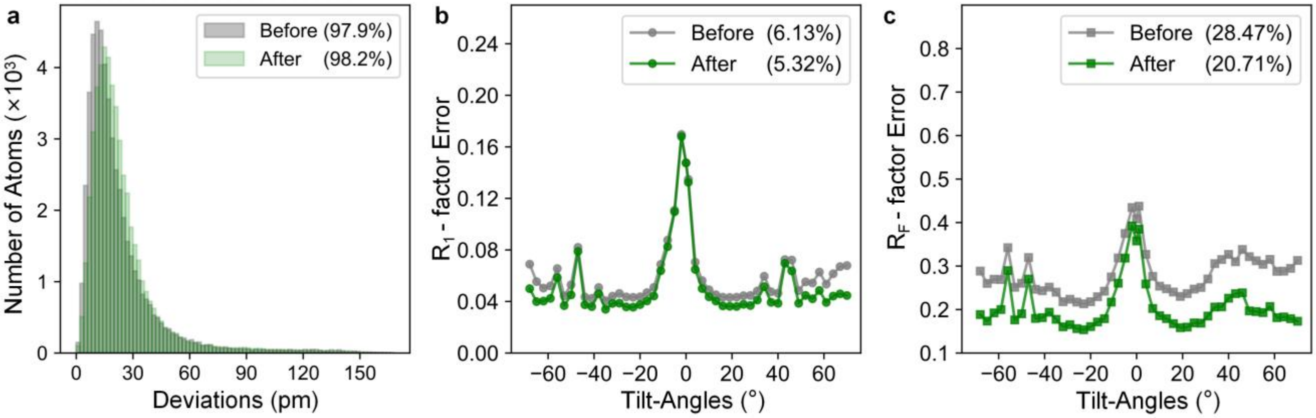

Fig. S19. Ablation study on defocus-corrected iterative refinement. a, Histogram of atomic deviations, with the common atomic ratio given in parentheses. Before and after iterative refinement with the 3D PSF, the RMSD values were 22.8 pm and 22.6 pm, respectively, with a difference of only 0.3% in the common atomic ratio. **b–c**, Real-space R_1-factor errors (**b**) and Fourier-space R_F-factor errors (**c**) before and after atomic structure refinement, with their average values in the legend.

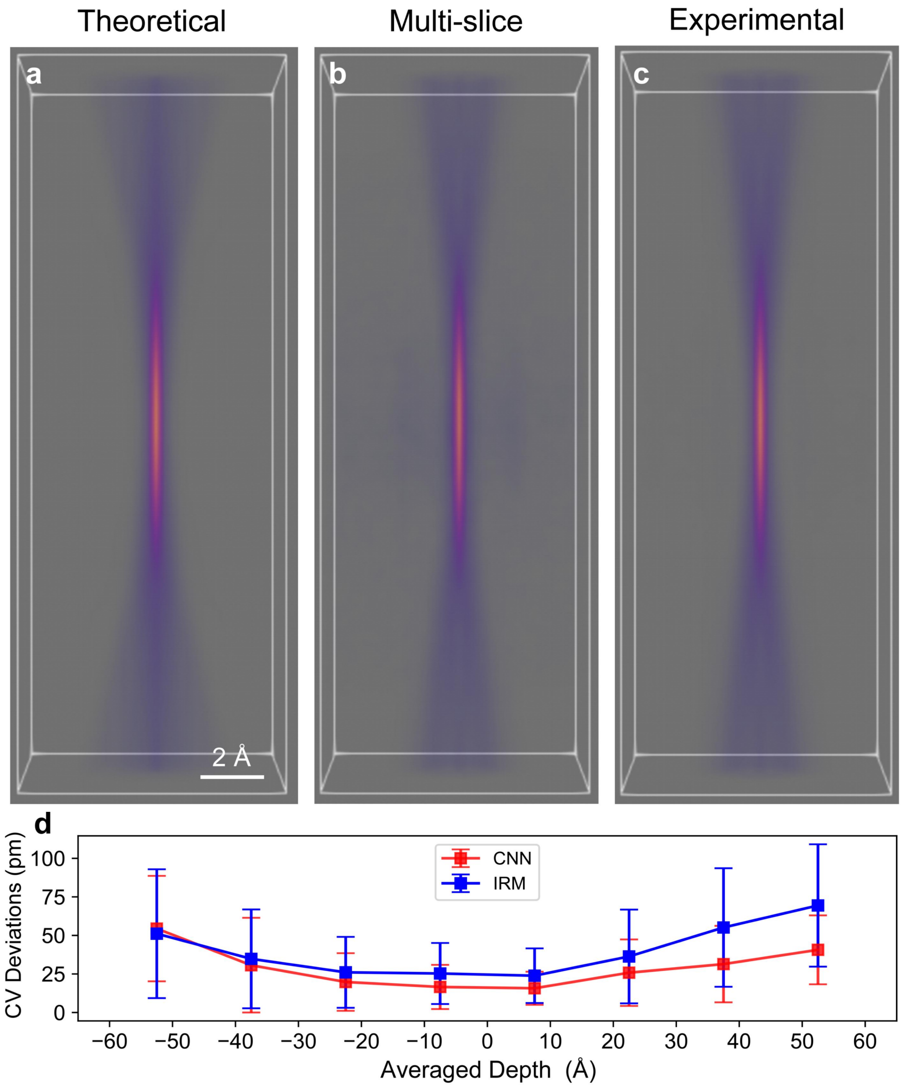

Fig. S20. Ablation study on multiple scattering. a, Theoretical PSF of ADF-STEM. **b,** Computed PSF fitted from the multi-slice simulated ADF-STEM image. **c,** Computed 3D PSF fitted from the experimental ADF-STEM image. **d,** Average atomic deviations and their standard deviations at different averaged depths estimated by cross-validation. Bins, 16 Å.

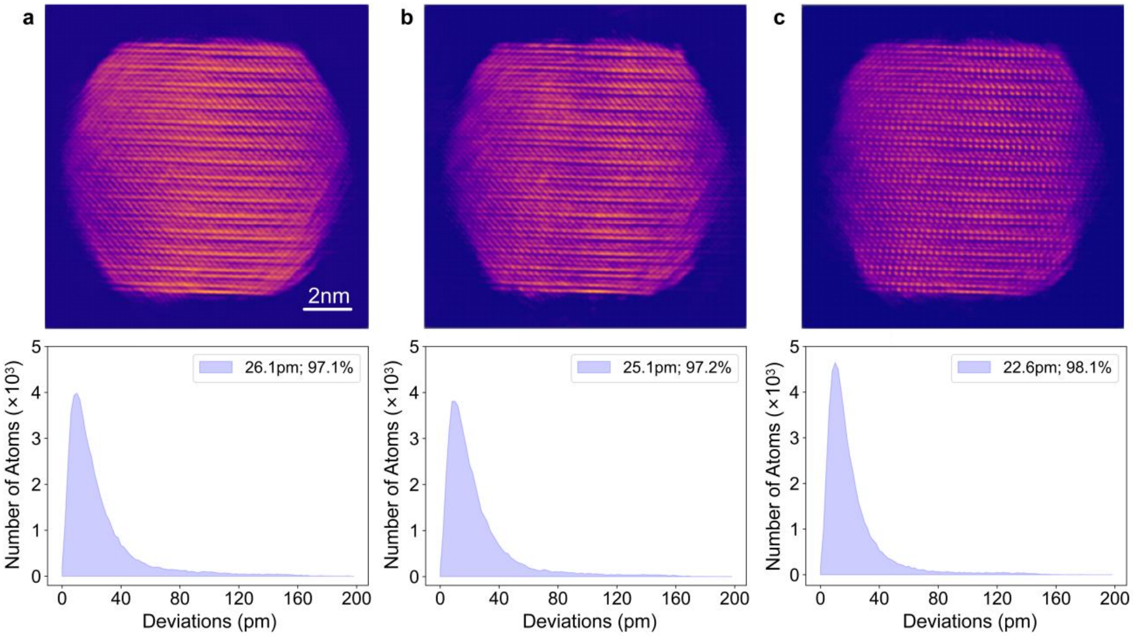

Fig. S21. Ablation study on training set size. a, CNN trained with5×10^3 data samples. **b,** CNN trained with5×10^4 data samples. **c,** CNN trained with5×10^5 data samples. The CNN-reconstructed tomogram (top) and the histogram of atomic deviations (bottom).

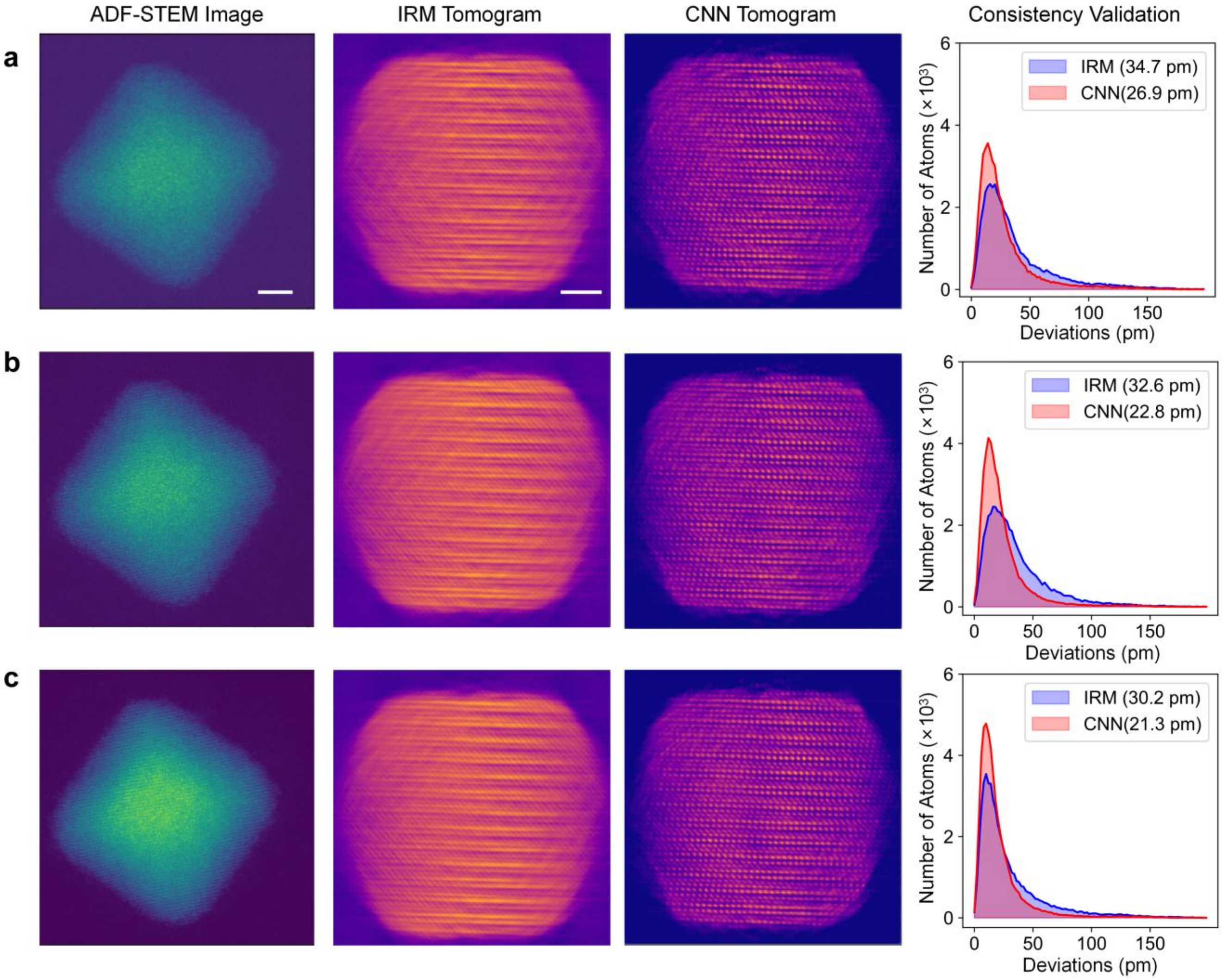

Fig. S22. Ablation study on electron dose. a, 0.62×10^5 e/Å². **b,** 1.24×10^5 e/Å². **c,** 1.87×10^5 e/Å². From left to right: Representative ADF-STEM image, IRM-reconstructed tomogram, CNN-reconstructed tomogram, histogram of atomic deviations. Scale bar, 2nm.

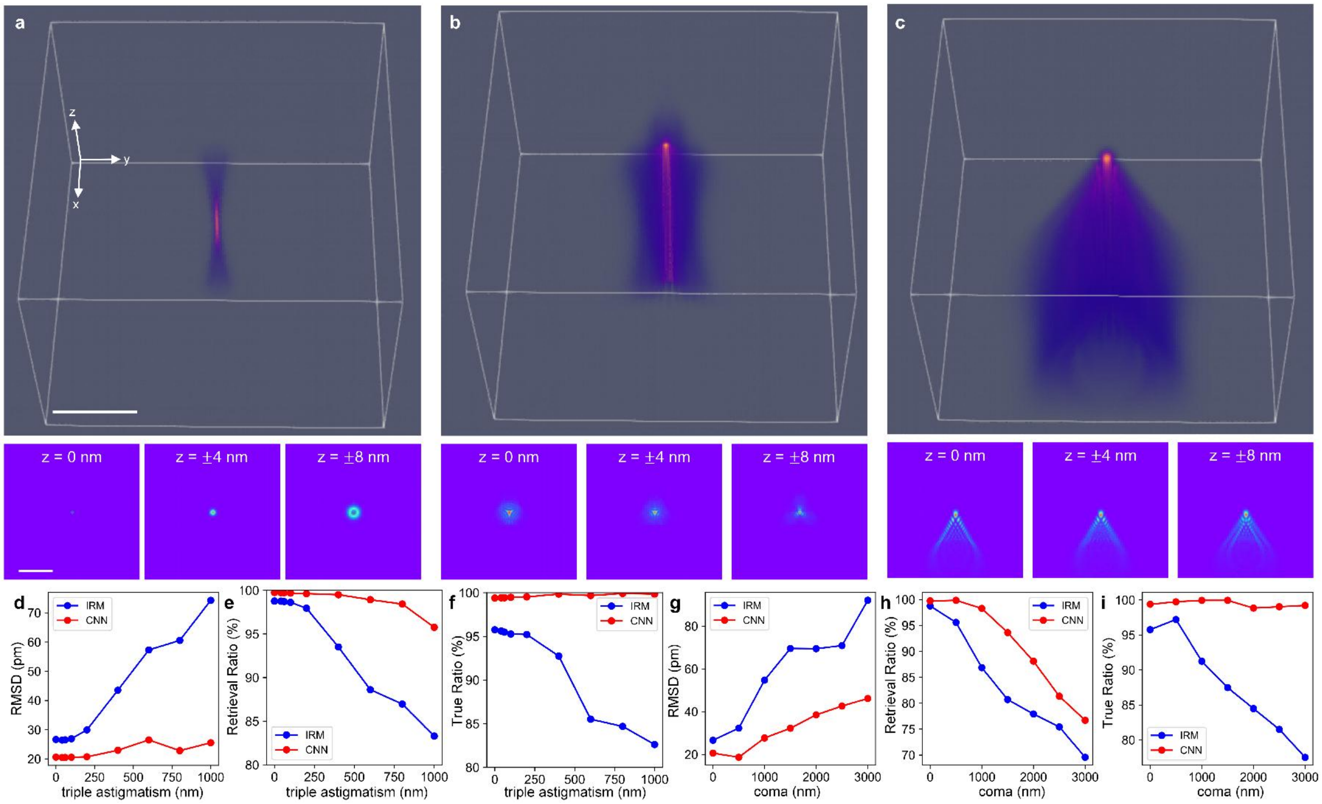

Fig. S23. Ablation study on anisotropic electron probe. a–c, Representative electron probe and its section at different depths. (a) contains only defocus, (b) contains defocus and triple astigmatism $A_3 = 1000\ nm$, and (c) contains defocus and coma $B_2 = 3000\ nm$. **d–f,** RMSD **(d)**, atomic retrieval ratio **(e)** and true atomic ratio **(f)** at different astigmatism values. **g–h,** RMSD **(g)**, atomic retrieval ratio **(h)** and true atomic ratio **(i)** at different coma values. Scale bar, 2nm.

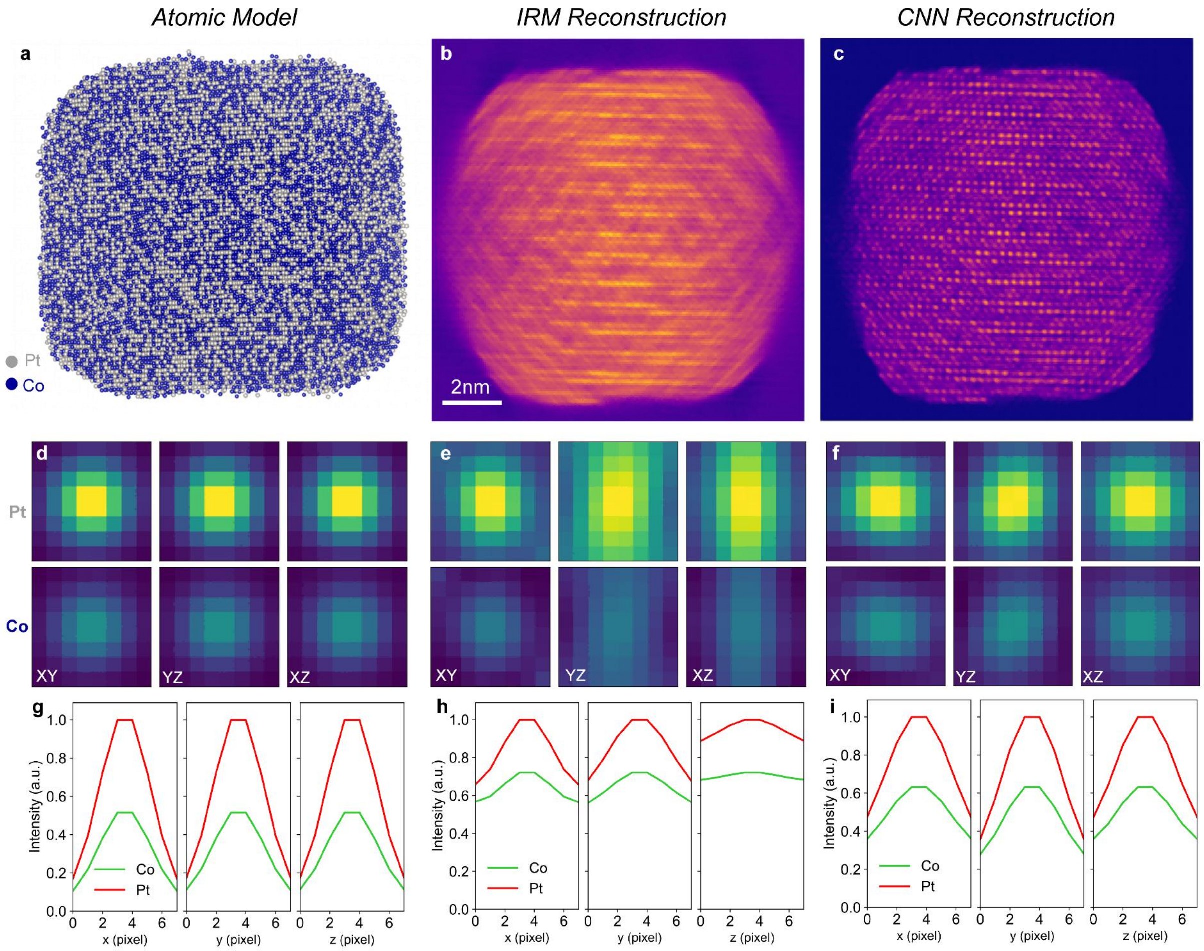

Fig. S24. Fidelity test of the contrast of the CNN model on a simulated PtCo tilt-series. a, Atomic model of PtCo alloy. **b–c,** Tomograms reconstructed by IRM (b) and augmented by CNN (c). **d–f,** 3D section of Pt and Co atoms, including the theoretical section obtained by convolving the Hartree-Fock atomic potential with PSF (d), averaged atomic sections fitted from the IRM-reconstructed tomogram (e) and the CNN-reconstructed tomogram (f). **g–i,** Radial atomic profiles of the theoretical atomic profile (g), IRM-reconstructed profile (h), and CNN-reconstructed profile (i).

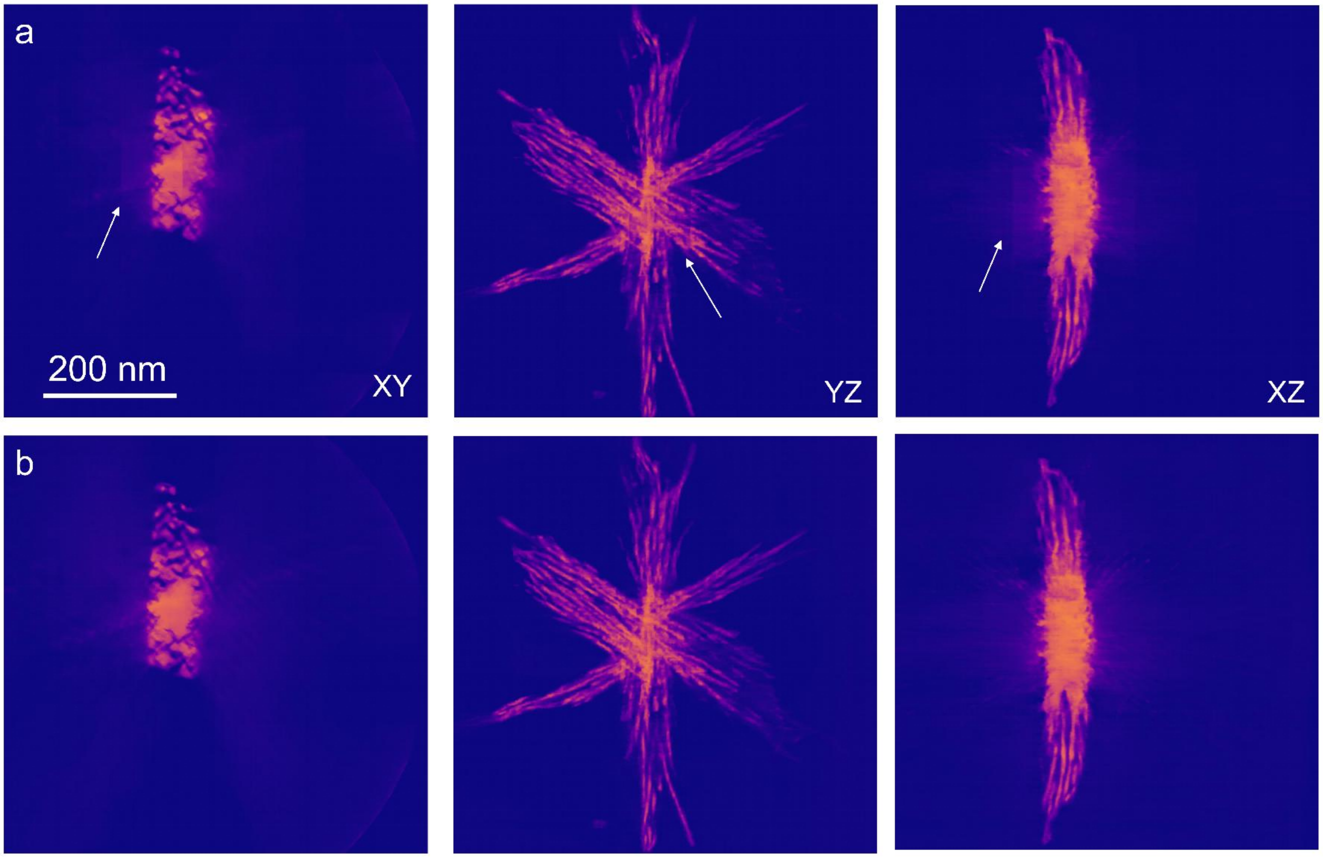

Fig. S25. Large-sized tomogram before and after the Gaussian fusion. a, Direct stitching of patches reconstructed by EM-CNN. **b,** Fused by Gaussian weights. From left to right: XY, YZ and XZ section.

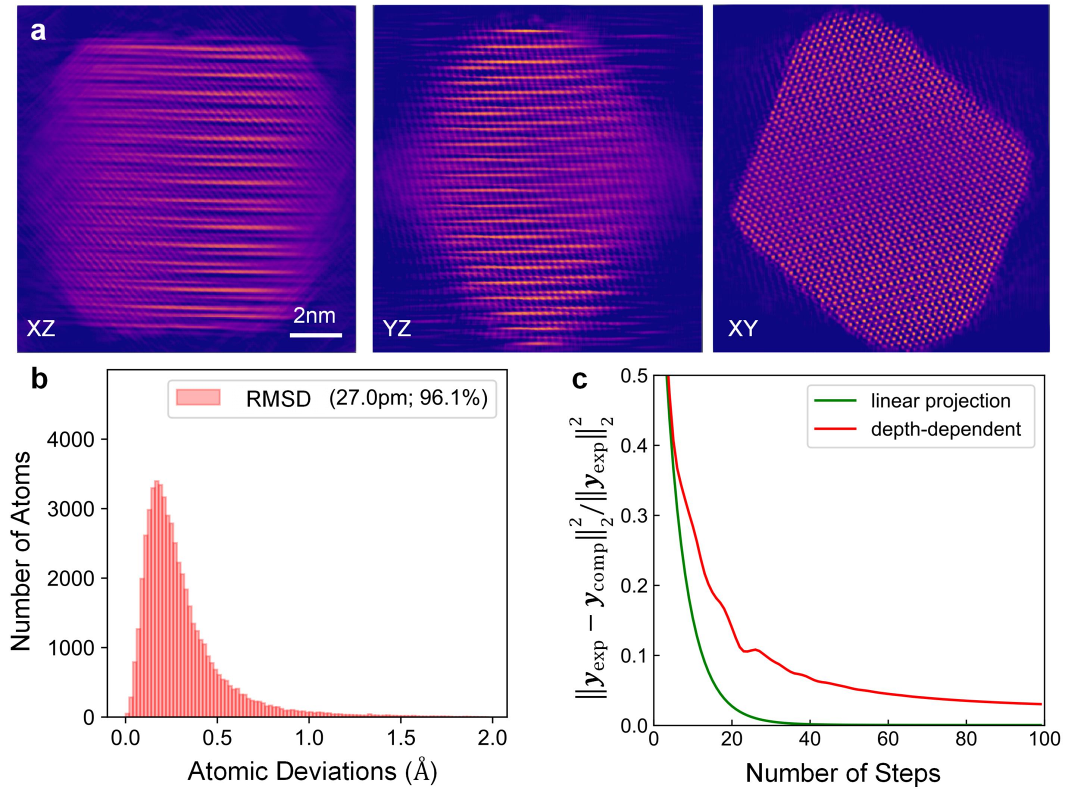

Fig. S26. Depth-dependent iterative reconstruction. a, Three orthogonal cross-sections of the tomogram reconstructed by the depth-dependent iterative reconstruction method. **b,** Histogram of atomic deviations. **c,** Optimization curves for linear projection and depth-dependent iterative reconstruction methods.

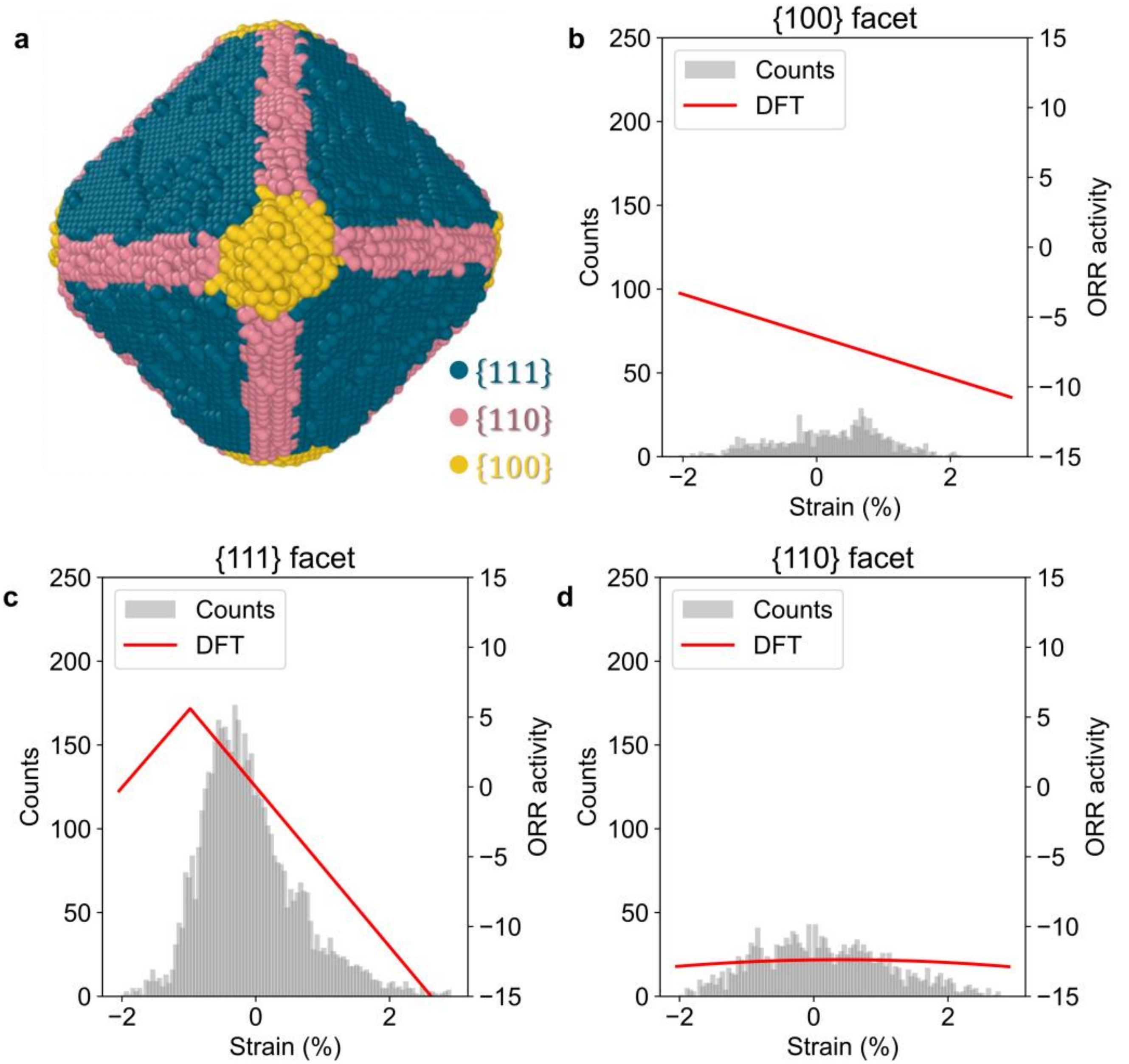

Fig. S27. Oxygen reduction reaction activity prediction of the 13-nm Pt nanoparticle reconstructed by CNN. a, Facet type of the CNN structure. **b–d,** Histograms of the surface atoms with different volumetric strain (gray), and the activity–strain curve predicted by density functional theory (red) in the literature[21, 34, 35].

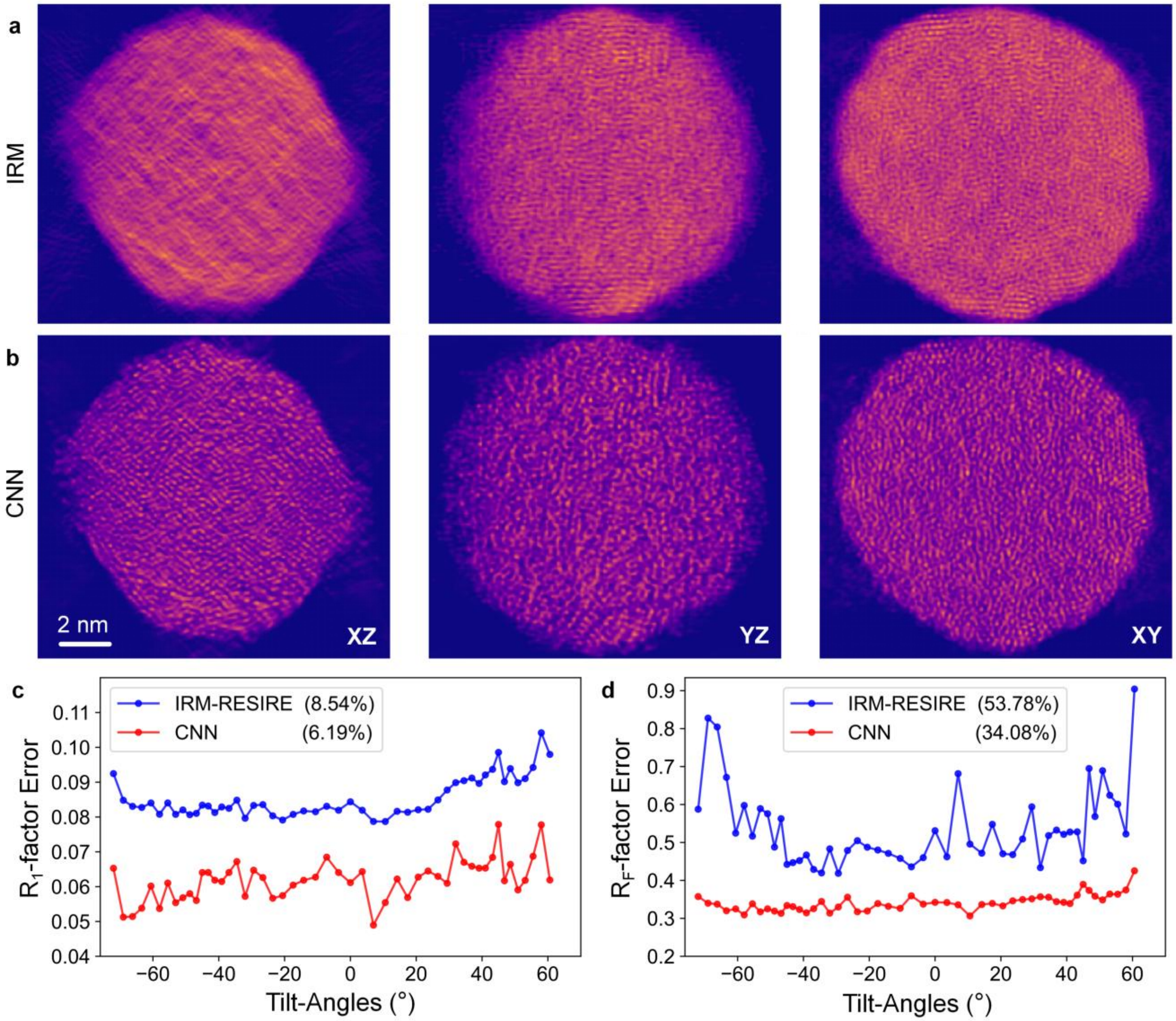

Fig. S28. Aperture corrected three-dimensional reconstruction of an amorphous Pd nanoparticle from publicly available datasets[18]. a, Three orthogonal cross-sections reconstructed by IRM, used as the input. **b,** Three orthogonal cross-sections after CNN augmentation. **c–d**, R_1-factor (**c**) and R_F-factor (**d**) of the reconstructed structures, the IRM errors were computed according to the reported structure. The amorphous Pd nanoparticle has a thickness of ~12 nm along the optical axis, which is basically within the depth-of-field of 17.1 mrad convergence semi-angle and 0.454 Å pixel size (Fig. S1g).

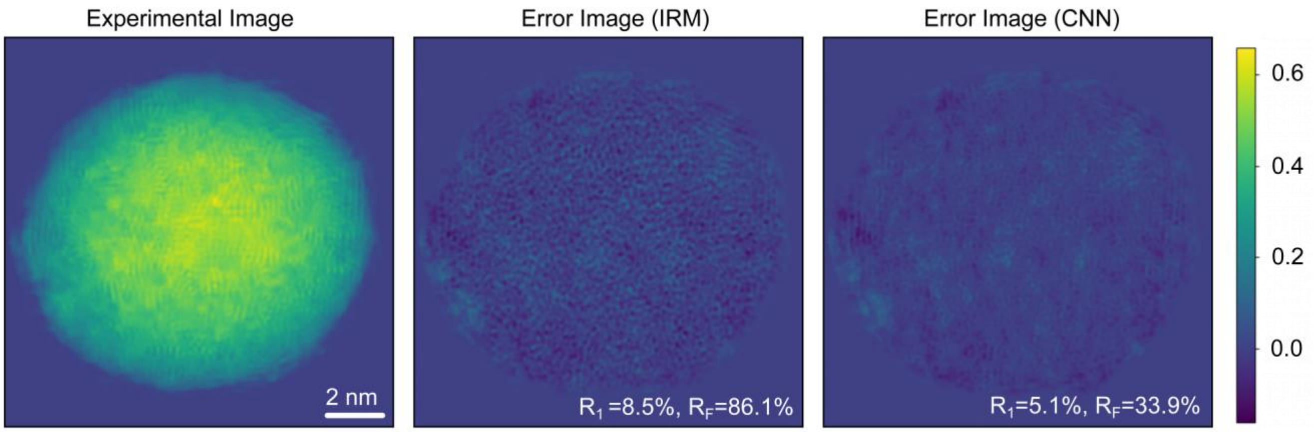

Fig. S29. Projection consistency validation of an amorphous Pd nanoparticle. From left to right: representative experimental image (-69°), error image of the RESIRE reconstructed structure reported in the literature[18], and the error image of the CNN-reconstructed structure. The information loss caused by low aperture will lead to a global resolution reduction, so the error is spatially uniformly distributed.

Tables

Table S1. Three-dimensional resolution of different methods on the validation dataset of 6° sparse projection tilt-series (estimated by Fourier shell correlation).

Dataset	Limit Resolution	IRM (Input)	EM-CNN	Tomo-CNN	ImgNet-CNN	FBP	TVM
Co_3O_4 Nanosheet	44.8 nm	219.5 nm	**49.9 nm**	129.1 nm	104.5 nm	365.9 nm	84.3 nm
MnO_2 Nanowire	31.7 nm	129.4 nm	**34.4 nm**	38.8 nm	452.8 nm	388.1 nm	349.3 nm
Hyperbranched Co_2P	14.2 Å	139.6 Å	**46.5 Å**	64.4 Å	59.8 Å	418.9 Å	119.7 Å
Nanoporous Carbon	13.95 Å	82.3 Å	**39.2 Å**	54.9 Å	45.7 Å	164.7 Å	102.9 Å
Nanoporous PtCu	7.6 Å	56.1 Å	**22.4 Å**	28.0 Å	34.5 Å	74.7 Å	32.0 Å

Table S2. Quantitative error of a multi-slice simulated Ta nanoparticle at 6° intervals.

Methods	RMSD (pm)	Common Atomic Ratio
EM-CNN	**11.9**	**98.4%**
Tomo-CNN	12.8	98.2%
ImgNet-CNN	13.7	97.8%
EM-CNN (without iterative refinement)	28.2	97.7%
Tomo-CNN (without iterative refinement)	15.0	98.2%
ImgNet-CNN (without iterative refinement)	42.7	96.7%
SART	20.0	95.1%
RESIRE	18.8	95.7%
GENFIRE	20.2	95.1%
TVM	29.4	90.4%

Table S3. Quantitative error of the disordered amorphous Pd particles collected under low convergence semi-angle (17.1 mrad).

Methods	Pd-1		Pd-2	
	R_1-factor Error	R_F-factor Error	R_1-factor Error	R_F-factor Error
CNN	**6.19%**	**34.08%**	**6.28%**	**28.31%**
IRM (RESIRE)	8.54%	53.78%	6.98%	44.06%
IRM (SART)	9.11%	43.64%	7.59%	43.64%
IRM (GENFIRE)	8.63%	42.36%	7.24%	43.16%

*Pd-1 is the particle shown in Figs.S28–29, containing 52,308 atoms, and Pd-2 is another particle reported in the same dataset[18], containing 76,238 atoms. The maximum thickness of both along the optical axis is ~12nm. Due to data scarcity, additional AET datasets for large-sized specimens are unavailable.

References

1. Kaczmarz S. Angenäherte auflösung von systemen linearer gleichungen (english translation by Jason Stockmann): Bulletin international de l'académie polonaise des sciences et des lettres. 1937.

2. Andersen AH, Kak AC. Simultaneous algebraic reconstruction technique (SART): a superior implementation of the ART algorithm. *Ultrason Imaging*. 1984; **6**(1): 81-94.

3. Levin BD, Padgett E, Chen C-C *et al.* Nanomaterial datasets to advance tomography in scanning transmission electron microscopy. *Sci Data*. 2016; **3**(1): 1-11.

4. Ronneberger O, Fischer P, Brox T. U-net: Convolutional networks for biomedical image segmentation. In: *Medical image computing and computer-assisted intervention–MICCAI 2015: 18th international conference, Munich, Germany, October 5-9, 2015, proceedings, part III 18, 2015*, p. 234-241. Springer.

5. DaCosta LR, Brown HG, Pelz PM *et al.* Prismatic 2.0–Simulation software for scanning and high resolution transmission electron microscopy (STEM and HRTEM). *Micron*. 2021; **151**: 103141.

6. Dabov K, Foi A, Katkovnik V *et al.* Image denoising by sparse 3-D transform-domain collaborative filtering. *IEEE Trans Image Process*. 2007; **16**(8): 2080-2095.

7. Makitalo M, Foi A. A closed-form approximation of the exact unbiased inverse of the Anscombe variance-stabilizing transformation. *IEEE Trans Image Process*. 2011; **20**(9): 2697-2698.

8. Otsu N. A threshold selection method from gray-level histograms. *Automatica*. 1975; **11**(285-296): 23-27.

9. Telea A. An image inpainting technique based on the fast marching method. *Journal of graphics tools*. 2004; **9**(1): 23-34.

10. Schwartz J, Harris C, Pietryga J *et al.* Real-time 3D analysis during electron tomography using tomviz. *Nat Commun*. 2022; **13**(1): 4458.

11. Girod R, Lazaridis T, Gasteiger HA *et al.* Three-dimensional nanoimaging of fuel cell catalyst layers. *Nat Catal*. 2023; **6**(5): 383-391.

12. Saxton W, Baumeister W. The correlation averaging of a regularly arranged bacterial cell envelope protein. *J Microsc*. 1982; **127**(2): 127-138.

13. Huang Y, Zhu C, Yang X *et al.* High-resolution real-space reconstruction of cryo-EM structures using a neural field network. *Nat Mach Intell*. 2024; **6**(8): 892-903.

14. Kucukelbir A, Sigworth FJ, Tagare HD. Quantifying the local resolution of cryo-EM density maps. *Nat Methods*. 2014; **11**(1): 63-65.

15. Yang Y, Chen C-C, Scott M *et al.* Deciphering chemical order/disorder and material properties at the single-atom level. *Nature*. 2017; **542**(7639): 75-79.

16. Zhou J, Yang Y, Yang Y *et al.* Observing crystal nucleation in four dimensions using atomic electron tomography. *Nature*. 2019; **570**(7762): 500-503.

17. Lee J, Jeong C, Yang Y. Single-atom level determination of 3-dimensional surface atomic structure via neural network-assisted atomic electron tomography. *Nat Commun*. 2021; **12**(1): 1962.

18. Yang Y, Zhou J, Zhu F *et al.* Determining the three-dimensional atomic structure of an amorphous solid. *Nature*. 2021; **592**(7852): 60-64.

19. Yuan Y, Kim DS, Zhou J *et al.* Three-dimensional atomic packing in amorphous solids with liquid-like structure. *Nat Mater*. 2022; **21**(1): 95-102.

20. Xu R, Chen C-C, Wu L *et al.* Three-dimensional coordinates of individual atoms in materials revealed by electron tomography. *Nat Mater*. 2015; **14**(11): 1099-1103.

21. Jo H, Wi DH, Lee T *et al.* Direct strain correlations at the single-atom level in three-dimensional core-shell interface structures. *Nat Commun*. 2022; **13**(1): 5957.

22. Moniri S, Yang Y, Ding J *et al.* Three-dimensional atomic structure and local chemical order of medium-and high-entropy nanoalloys. *Nature*. 2023; **624**(7992): 564-569.
23. Li Z, Xie Z, Zhang Y *et al.* Probing the atomically diffuse interfaces in Pd@ Pt core-shell nanoparticles in three dimensions. *Nat Commun*. 2023; **14**(1): 2934.
24. Hong J, Bae J-H, Jo H *et al.* Metastable hexagonal close-packed palladium hydride in liquid cell TEM. *Nature*. 2022; **603**(7902): 631-636.
25. Yang Y, Zhou J, Zhao Z *et al.* Atomic-scale identification of active sites of oxygen reduction nanocatalysts. *Nat Catal*. 2024; **7**(7): 796-806.
26. Chen C-C, Zhu C, White ER *et al.* Three-dimensional imaging of dislocations in a nanoparticle at atomic resolution. *Nature*. 2013; **496**(7443): 74-77.
27. Scott M, Chen C-C, Mecklenburg M *et al.* Electron tomography at 2.4-ångström resolution. *Nature*. 2012; **483**(7390): 444-447.
28. Tian X, Kim DS, Yang S *et al.* Correlating the three-dimensional atomic defects and electronic properties of two-dimensional transition metal dichalcogenides. *Nat Mater*. 2020; **19**(8): 867-873.
29. Zhang H, Ha D-H, Hovden R *et al.* Controlled synthesis of uniform cobalt phosphide hyperbranched nanocrystals using tri-n-octylphosphine oxide as a phosphorus source. *Nano Lett*. 2011; **11**(1): 188-197.
30. Jeong C, Lee J, Jo H *et al.* Atomic-scale 3D structural dynamics and functional degradation of Pt alloy nanocatalysts during the oxygen reduction reaction. *Nat Commun*. 2025; **16**(1): 8026.
31. Jeong C, Lee J, Jo H *et al.* Revealing the three-dimensional arrangement of polar topology in nanoparticles. *Nat Commun*. 2024; **15**(1): 3887.
32. Reshef DN, Reshef YA, Finucane HK *et al.* Detecting novel associations in large data sets. *Science*. 2011; **334**(6062): 1518-1524.
33. Zhang Y, Cao L, Sun Z *et al.* Physics-aware neural networks enable robust and full atomic structure determination via low-dose atomic electron tomography. *arXiv preprint arXiv:260319942*. 2026.
34. Lee J, Jeong C, Lee T *et al.* Direct observation of three-dimensional atomic structure of twinned metallic nanoparticles and their catalytic properties. *Nano Lett*. 2022; **22**(2): 665-672.
35. Viswanathan V, Hansen HA, Rossmeisl J *et al.* Universality in oxygen reduction electrocatalysis on metal surfaces. *ACS Catal*. 2012; **2**(8): 1654-1660.
36. Liu Y-T, Zhang H, Wang H *et al.* Isotropic reconstruction for electron tomography with deep learning. *Nat Commun*. 2022; **13**(1): 6482.
37. Kim NY, Zhong H, Zhang J *et al.* Three-dimensional imaging of individual carbon atoms. *arXiv preprint arXiv:250408228*. 2025.
38. Mao L, Cui J, Yu R. Local-orbital tomography with depth-dependent interactions. *Phys Rev B*. 2025; **111**(6): 064116.
39. Mao L, Cui J, Yu R. 3D reconstruction of a million atoms by multiple-section local-orbital tomography. *Sci Bull*. 2025; **70**(1): 64-69.
40. Larsen PM, Schmidt S, Schiøtz J. Robust structural identification via polyhedral template matching. *Modell Simul Mater Sci Eng*. 2016; **24**(5): 055007.
